# Direct Keap1-Nrf2 disruption as a potential therapeutic target for Alzheimer’s disease

**DOI:** 10.1371/journal.pgen.1006593

**Published:** 2017-03-02

**Authors:** Fiona Kerr, Oyinkan Sofola-Adesakin, Dobril K. Ivanov, Jemma Gatliff, Beatriz Gomez Perez-Nievas, Hélène C. Bertrand, Pedro Martinez, Rebecca Callard, Inge Snoeren, Helena M. Cochemé, Jennifer Adcott, Mobina Khericha, Jorge Iván Castillo-Quan, Geoffrey Wells, Wendy Noble, Janet Thornton, Linda Partridge

**Affiliations:** 1 Institute of Healthy Ageing, and GEE, University College London, Darwin Building, Gower Street, London, United Kingdom; 2 European Molecular Biology Laboratory, European Bioinformatics Institute, Wellcome Trust Genome Campus, Hinxton, Cambridge, United Kingdom; 3 UCL School of Pharmacy, University College London, 29/39 Brunswick Square, London, United Kingdom; 4 Maurice Wohl Clinical Neuroscience Institute, King’s College London, London, United Kingdom; 5 Max Planck Institute for Biology of Ageing, Köln, Germany; 6 MRC London Institute of Medical Sciences, Imperial College London, Du Cane Road, London, United Kingdom; Stanford University School of Medicine, UNITED STATES

## Abstract

Nrf2, a transcriptional activator of cell protection genes, is an attractive therapeutic target for the prevention of neurodegenerative diseases, including Alzheimer’s disease (AD). Current Nrf2 activators, however, may exert toxicity and pathway over-activation can induce detrimental effects. An understanding of the mechanisms mediating Nrf2 inhibition in neurodegenerative conditions may therefore direct the design of drugs targeted for the prevention of these diseases with minimal side-effects. Our study provides the first *in vivo* evidence that specific inhibition of Keap1, a negative regulator of Nrf2, can prevent neuronal toxicity in response to the AD-initiating Aβ42 peptide, in correlation with Nrf2 activation. Comparatively, lithium, an inhibitor of the Nrf2 suppressor GSK-3, prevented Aβ42 toxicity by mechanisms independent of Nrf2. A new direct inhibitor of the Keap1-Nrf2 binding domain also prevented synaptotoxicity mediated by naturally-derived Aβ oligomers in mouse cortical neurons. Overall, our findings highlight Keap1 specifically as an efficient target for the re-activation of Nrf2 in AD, and support the further investigation of direct Keap1 inhibitors for the prevention of neurodegeneration *in vivo*.

## Author summary

As our population ages the incidence of neurodegenerative diseases, including Alzheimer’s disease (AD), is predicted to increase dramatically. Despite providing important symptomatic relief, existing treatments for such conditions do not slow-down disease progression, and this will cause an overwhelming future burden on our healthcare system and immense suffering for many more patients and their families. Nrf2 is a gene that normally protects cells from stressful conditions. Although we don’t know why, Nrf2 is reduced in the brains of AD patients and this may explain the increased susceptibility of neurons to damage in neurodegenerative diseases. Our research, using a fruit fly model, identifies Keap1, a negative regulator of Nrf2, as a valid target for the rescue of AD-related Nrf2 defects and the subsequent prevention of neuronal degeneration. Moreover, we show that a new compound, which directly blocks the binding between Nrf2 and Keap1, can prevent toxicity of the AD-initiating Aβ peptide in mouse neurons. Hence, our study provides strong evidence that direct Keap1-Nrf2 disruptors can specifically target the defects in Nrf2 activity observed in neurodegenerative diseases, and supports the further development of such compounds as potential new drugs to prevent neuronal decline AD and other neurodegenerative conditions.

## Introduction

The transcription factor Nrf2 (nuclear factor E2-related factor 2) targets cellular defence genes containing antioxidant response elements (ARE), which include antioxidant enzymes (glutamate cysteine ligase; GCL), drug metabolising enzymes (cytochrome P450s, glutathione S-transferases; GSTs), molecular chaperones, DNA repair enzymes and proteasome subunits[1]. Activation of these protective genes in response to Nrf2 enables the cell to maintain redox balance and to remove damaged proteins under conditions of oxidative and xenobiotic stress.

Such cellular stress is a key feature of several neurodegenerative diseases. Markers of oxidative damage are increased in the brains of Alzheimer’s disease (AD)[2,3], Parkinson’s disease (PD)[4–6], Huntington’s disease (HD)[7] and, in CSF, of Amyotrophic Lateral Sclerosis (ALS) patients[8]. Some evidence also suggests that AD, PD and ALS patients have reduced xenobiotic metabolism [9] and that the AD-causing Aβ42 peptide may act as a xenobiotic[10]. As Nrf2 is inhibited in several neurodegenerative diseases[1, 11], including AD[11] and ALS[12], as well as in APP/PS1 mutant mouse models of AD[13], deficits in this important cellular protection pathway may, in part, explain the cellular damage associated with these conditions. Conversely, Nrf2 over-expression protects against toxicity induced by the Aβ42 peptide in mammalian cells[13,14], and prevents neuronal pathology in mouse models of ALS[15], PD[16] and AD[17]. Activation of Nrf2 is, therefore, increasingly implicated as an attractive target for the prevention of several neurodegenerative conditions.

Potent activators of Nrf2 have been developed[18], and confer protection against chemically-induced neurotoxic insults[19] and improve memory deficits in mouse models of AD[20]. Many of these Nrf2 activators, however, have been reported to exert toxicity due to off-target effects[21][22]. Additionally, unregulated activation of Nrf2 can have detrimental consequences, with prolonged, ubiquitous activation shortening lifespan in *Drosophila*[23], and mutations in the Nrf2 inhibitor Keap1 (kelch-like ECH-associating protein 1) causing cancer in humans[24]. Hence, a better understanding of the mechanisms by which Nrf2 function is inhibited in neurodegenerative disease may enable the design of drugs targeted for the prevention of these conditions with minimal side-effects.

Nrf2 activity is tightly regulated by two main inhibitors, Keap1 and GSK-3 (glycogen synthase kinase-3)[25] (S1 Fig). GSK-3 plays a well-established role in the pathogenesis of AD[26], and GSK-3 inhibitors, including lithium, prevent pathology in animal models of AD[26,27], ALS[28] and PD[29]. Emerging evidence also suggests that inhibiting Keap1 can ameliorate neuronal degeneration, with Keap1 RNA interference (RNAi) protecting against Aβ42[30] and MPTP[31] toxicity in cells, and heterozygous loss of Keap1 protecting against neuronal pathology in *Drosophila* models of PD[32,33]. Both GSK-3 and Keap1 may, therefore, serve as valid candidates for mediating the inhibition of Nrf2 in neurodegenerative diseases, and their inhibition may enable the prevention of Nrf2 deficits, neuronal stress and degeneration specifically in these conditions. A comparative analysis of the efficiency of their inhibition in rescuing Nrf2 deficits in neurodegenerative diseases specifically, however, is required.

We aimed therefore to investigate the role of Nrf2 in mediating the protective effects of GSK-3 and Keap1 inhibition against Aβ42 toxicity. Using an inducible *Drosophila* model of AD[34], we confirmed that Aβ42 inhibits activity of the fly homolog of Nrf2 (cap-n-collar isoform C, cncC[35]) in neurons, consistent with previous findings in mice[13]. Both inhibition of GSK-3, using lithium, and loss-of-function mutations in Keap1 protected against Aβ42 toxicity in this model. We found, however, that neuronal protection in response to Keap1 inhibition correlates with the rescue of Aβ42-induced Nrf2 defects, whereas lithium treatment appears to exert neuro-protection independently of Nrf2.

Consistent with Nrf2 activation, Keap1 inhibition prevented the enhanced sensitivity of Aβ42-expressing flies to xenobiotic stress, but exerted minimal protection against oxidative damage in comparison to lithium treatment. Combined modulation of Keap1 and lithium additively protected against Aβ42 toxicity, in comparison with either treatment alone, but did not improve their respective effects on xenobiotic and oxidative damage. This further supports the divergent beneficial effects of these manipulations. Down-regulation of Keap1 alone additionally protected against Aβ42 toxicity by mechanisms correlating with enhanced degradation of Aβ42 peptide.

Overall our data highlight Keap1 as an efficient target for the amelioration of Nrf2 deficits and protection against neuronal damage in AD. Finally, we show for the first time that a newly-described direct inhibitor of the Keap1-Nrf2 protein-protein interaction[22] can indeed protect against the synapto-toxicity of naturally-derived Aβ oligomers in primary mouse neuronal cultures. As current Nrf2 activators may exert toxicity due to off-target effects, our data suggest that blocking the specific interaction of Nrf2 with Keap1 may provide an exciting new avenue for the discovery of disease-modifying treatments for AD, and potentially other neurodegenerative conditions.

## Results

### Aggregating Aβ42 peptides inhibit Nrf2/cncC activity in *Drosophila*

**Fig 1:**
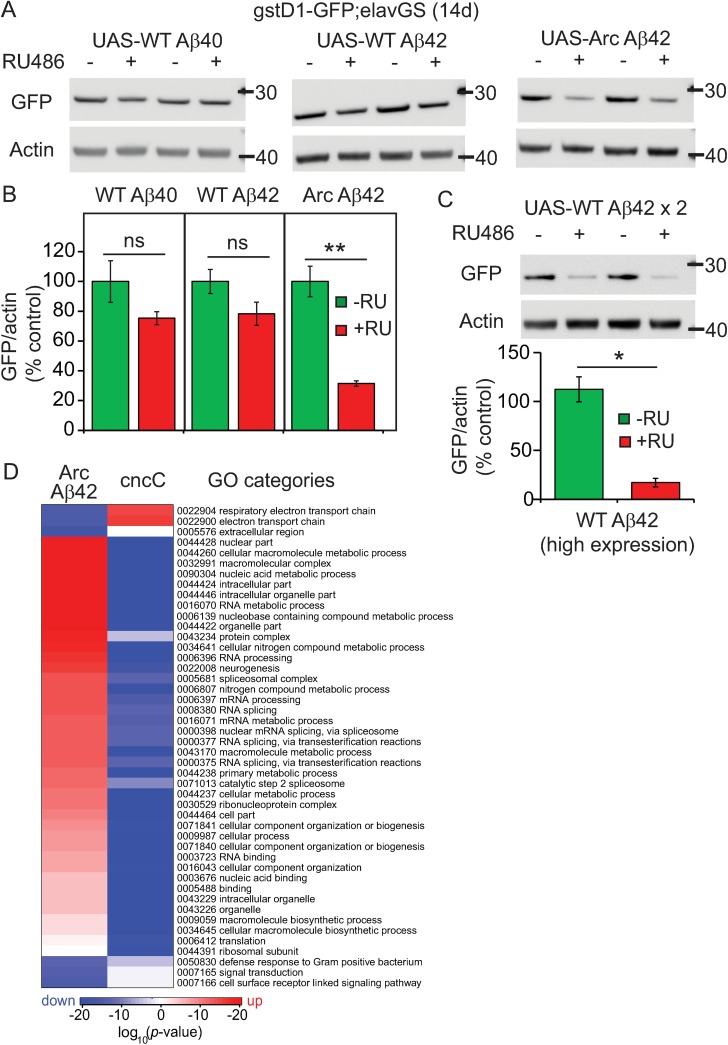
Aβ42 inhibits cncC activity in *Drosophila*. (A) Nrf2/cncC activity was measured by crossing *gstD1*(ARE)–GFP reporter flies to UAS-attP Aβ-expressing lines, under control of the *Drosophila* necrotic signal peptide [36], then measuring GFP expression in heads by Western blotting. (B) Quantitation of WB depicted in (A) above. A separate experiment was run for each Aβ-expressing line. GFP expression was normalized to actin, for–RU and +RU samples, then expressed as a percentage of the average–RU value for each blot to enable comparison. ArcAβ42 significantly reduced GFP expression compared to controls (** *p*< 0.01 comparing +RU to–RU). *p*>0.05 comparing +RU to–RU for WT Aβ40 and 42 lines (student’s t-test). Data are presented as means ± SEM and were analysed by student’s t-test for each genotype. N = 4 biological repeats of 10 fly heads per sample. (C) Analysis of Nrf2/cncC activity in response to high levels of WT Aβ42 expression. *gstD1*(ARE)–GFP reporter flies were crossed to flies expressing a tandem double copy of WT Aβ42 under the control of the argos signal peptide [37]. Data were analysed as described in (B). * *p*<0.05 comparing -RU to +RU (student’s t-test). N = 4 biological repeats of 10 fly heads per sample. (D) Heatmaps depicting gene ontology (GO) categories altered by ArcAβ42 expression in + RU vs–RU UAS-Arc Aβ42>elavGS flies (column Arc Aβ42) and that overlap with categories altered by cncC over-expression[38]; column cncC). Red indicates higher expression; blue indicates lower expression (scale = log_10_ fold change). A random insertion UAS-ArcAβ42 line was used for these experiments[39]. See S1A Fig for effects of ArcAβ42 induction of Nrf2 activity in heads and bodies using these flies. See S1B Fig for Venn diagrams of reciprocal effects of ArcAβ42 and cncC activation on GO category expression.

Over-expression of APP in mice inhibits expression of Nrf2 target genes[13]. Comparing expression, at equivalent levels[36], of various Aβ species in adult fly neurons, we have shown that the presence of aggregating Aβ42 peptides may specifically mediate this effect. Using Nrf2/cncC reporter flies (*gstD1*-GFP; [35]), RU486 induction of a single, site-directed, copy of Arctic mutant Aβ42 (ArcAβ42), but not WT Aβ40 or Aβ42 peptides, significantly reduced Nrf2/cncC activity compared to un-induced *gstD1*-GFP-expressing controls (Fig 1A and 1B). Only ArcAβ42 flies develop pathological phenotypes under these expression conditions. Moreover, inducing high levels of WT Aβ42 using an independent random-insertion line, which does cause neuronal decline [37], also significantly reduced Nrf2/cncC activity (Fig 1C). This suggests that the inhibition of Nrf2 may be related to the concentration-dependent ability of Aβ42 to aggregate and exert toxicity.

Consistent with a specific inhibition of neuronal Nrf2/cncC activity in response to Aβ42 expression, ArcAβ42 reduced GFP levels in the heads, but not bodies (S2A Fig), of RU486-induced flies. Microarray analyses further revealed several pathways and processes that might mediate toxicity in response to ArcAβ42 expression in fly neurons (Fig 1D, Arc Aβ42). Comparing this data-set to a previously published microarray analysis using flies ubiquitously over-expressing Nrf2/cncC (Fig 1D, cncC[38]), we found that Aβ42 expression induced reciprocal effects on gene ontology (GO) categories normally regulated by the Nrf2 pathway. Aβ42 activated processes that are down-regulated, and inhibited processes that are up-regulated, by cncC (Fig 1D & S2B Fig), further confirming its suppressive effect on Nrf2 signalling.

Overall these findings suggest that the inhibition of Nrf-2 in AD is robustly conserved in *Drosophila*, and that this deficiency is caused by the Aβ42 peptide directly. Moreover, as we also observed a suppression of Nrf2/cncC in flies over-expressing human 0N3R tau or the ALS-related C9orf72 (GR) 100 di-peptide repeat protein (DPR) [40] in adult neurons (S3A and S3B Fig), our findings suggest that the accumulation of toxic proteins may lead to the generalised defect in Nrf2 signalling observed in neurodegenerative diseases.

### At therapeutic levels inhibition of Keap1, but not lithium treatment, rescues Nrf2 defects in response to Aβ42 expression

*Drosophila* thus provide an excellent context for further analysis of the mechanisms by which Aβ42 regulates Nrf2 *in vivo*. As cncC, MafS and Keap1 mRNA transcripts were unaltered by Aβ42 in our flies (S3C Fig), we hypothesised that Aβ42 modulates Nrf2 activity by altering its biochemical interaction with upstream regulators.

**Fig 2:**
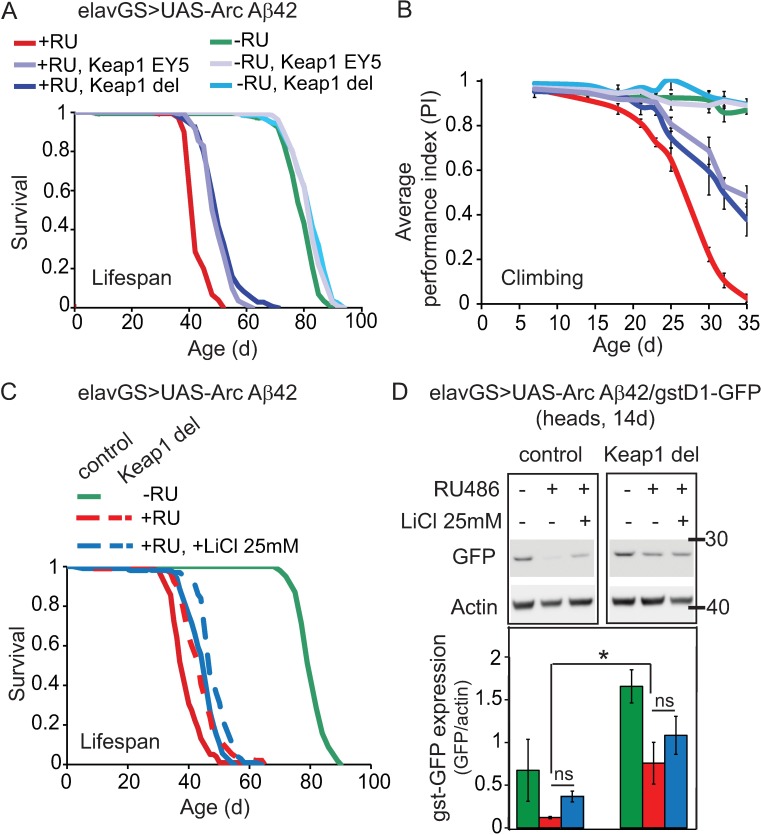
Keap1 reduction, but not lithium treatment, protects against Aβ42 toxicity in correlation with increased cncC activity. (A) Heterozygous loss of Keap1 extended lifespan of ArcAβ42-expressing flies. *P* = 0.001 comparing +RU, Keap1 del or +RU, Keap1 EY5 flies to +RU alone (log-rank test). N = 150 flies per condition. (B) Heterozygous loss of Keap1 ameliorated climbing deficiency in ArcAβ42 flies. *P*<0.05 comparing +RU, Keap1 del or +RU Keap1 EY5 flies to +RU alone (two-way ANOVA). N = 45–60 flies per condition analysed as 3–4 biological repeats of 15 flies. (C) Lifespan extension of ArcAβ42-expressing flies by lithium (*p*<0.001 comparing +RU to +RU, +LiCl 25 mM) was further extended by reducing Keap1 levels. *P*<0.001 comparing +RU + LiCl 25 mM to +RU + LiCl 25 mM, Keap1 del. Keap1 del and 25 mM lithium alone protected against Aβ42 toxicity to the same extent. *P* = 0.739 comparing +RU, + LiCl 25 mM to +RU, Keap1 del (log-rank test). N = 100 flies per condition. (D) cncC activity, as measured using a *gstD1*-GFP reporter, in ArcAβ42-expressing flies treated with lithium in combination with heterozygous loss of Keap1. Data are presented as means ± SEM and were analysed by two-way ANOVA followed by Tukey’s post-hoc analyses. *P*<0.001 comparing Keap1 del to control flies (two-way ANOVA). *P*<0.05 comparing +RU to +RU, Keap1 del. *P*>0.05 comparing +RU to +RU, +LiCl 25 mM and +RU, Keap1 del to +RU, Keap1 del + LiCl 25 mM. N = 4 replicates of 10 flies per condition.

Keap1 is a well-known negative regulator of Nrf2 activity in response to oxidative and xenobiotic stressors[41], but its role in mediating damage in response to neurotoxic proteins has not been widely studied *in vivo*. Genetically reducing Keap1, alone, extended lifespan (Fig 2A) and rescued neuronal-specific motor defects in both WT (S4 Fig) and ArcAβ42-expressing flies (Fig 2B) using two independent alleles, Keap1^del^ [42] and Keap1^EY5^[35]. GSK-3 has more recently been shown to inhibit Nrf-2[43], independently of Keap1, but has long been implicated in the pathogenesis of several neurodegenerative diseases including AD[26]. We have previously shown that lithium, a well-described GSK-3 inhibitor[44–46], prevents Aβ42 toxicity in our fly model [34]. Thus both GSK-3 and Keap1 may serve as effective targets for the prevention of neurodegeneration in AD.

We next compared the effects of manipulating both Keap1 and GSK-3 regulatory pathways on Aβ42 toxicity and Nrf2/cncC activity in a parallel study. Using a dose of lithium (25 mM) that prevented Aβ42-induced lifespan-shortening to a similar extent as heterozygous loss-of-function mutations in Keap1 (Fig 2C, *P* = 0.739 comparing +RU, + LiCl 25 mM to +RU, Keap1 del), this comparative analysis revealed that both manipulations rescued lifespan-shortening in Aβ42-expressing flies in an additive manner (Fig 2C, *P*<0.001 comparing +RU + LiCl 25 mM to +RU + LiCl 25 mM, Keap1 del), consistent with an independent mechanism of action.

At these therapeutic levels, genetically reducing Keap1, alone, significantly rescued the decline in Nrf2/cncC reporter expression observed in Aβ42-expressing flies (Fig 2D, *p*<0.05 comparing +RU vs +RU, Keap1 del). By contrast, although 25 mM lithium was sufficient to rescue Aβ42 toxicity, a dose of 50–100 mM lithium, which may exert toxic effects [47], was required to rescue Nrf2/cncC activity as measured using both microarray (S5A Fig) and gstD2 expression (S5B Fig) analyses. A heterozygous loss-of-function mutation in cncC (cncCK6[48]; S5C Fig) did not alter the ability of lithium to protect against Aβ42-mediated lifespan-shortening, further suggesting that Nrf2 is not required for lithium to exert its protective effects *in vivo*. Consistent with our previous finding that lithium reduces Aβ42 levels, and prevents toxicity, by blocking translation[47], early intervention concurrent with RU486 induction was required for lithium to protect against Aβ42-induced climbing defects (S5D Fig) and was correlated with reduced Aβ42 levels from the point of induction (S5E Fig). This suggests that, at therapeutically active concentrations, the protective effect of lithium against Aβ42 toxicity is not predominantly mediated by activating Nrf2, but rather by blocking Aβ42 peptide accumulation through inhibition of translation.

Our data provide the first evidence that specific Keap1 inhibition can protect against Aβ42 toxicity *in vivo*, and they highlight Keap1 as a more efficient target for the prevention of Aβ42 peptide-induced Nrf2 inhibition in comparison with lithium treatment.

### Keap1 inhibition protects predominantly against Aβ42-induced xenobiotic, not oxidative, damage

**Fig 3:**
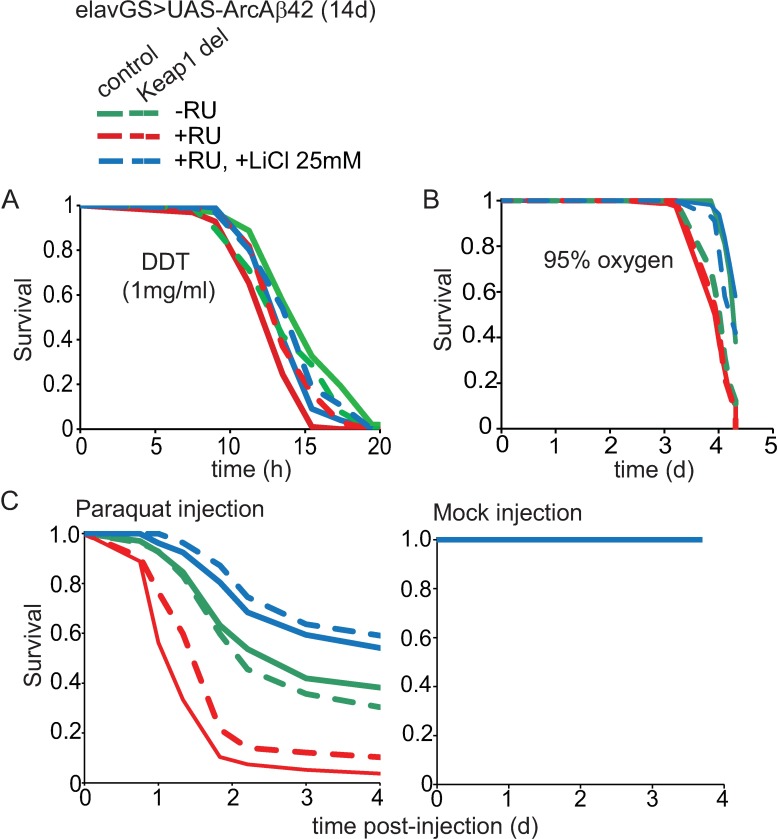
Effects of Keap1 inhibition or lithium treatment on sensitivity of Aβ42 flies to oxidative and xenobiotic stress. (A) Keap1 deletion and lithium treatment combined to protect against sensitivity to xenobiotic damage in Aβ42-expressing flies (*p*<0.001 comparing–RU to +RU). Chronic lithium treatment and loss of Keap 1 alone protected against Aβ42-induced DDT sensitivity to the same extent. *P*<0.001 comparing +RU to +RU + LiCl 25 mM and +RU, Keap1 del flies. *P* = 0.886 comparing +RU +LiCl 25 mM to +RU, Keap1 del flies. Combined lithium treatment and Keap1 inhibition protected Aβ42 flies against DDT toxicity to a greater extent than lithium treatment, but not Keap1 deletion, alone. *P*<0.05 comparing +RU + LiCl 25mM to +RU + LiCl 25 mM, Keap1 del. *P* = 0.057 comparing +RU, Keap1 del to +RU + LiCl 25 mM, Keap1 del (log-rank test). N = 100 flies per condition. (B) Lithium treatment, but not Keap1 inhibition, protected against Aβ42-induced sensitivity to hyperoxia (95% oxygen; *p*<0.001 comparing–RU to +RU). Lithium treatment alone significantly ameliorated sensitivity to hyperoxia (*p*<0.05 comparing +RU to +RU + LiCl 25 mM), but heterozygous loss of Keap1 alone did not (*p* = 0.99 comparing +RU to +RU, Keap1 del). Keap1 deletion prevented, rather than enhanced, the protective effect of lithium against Aβ42-induced sensitivity (*p*<0.01 comparing +RU + LiCl 25 mM to +RU + LiCl 25 mM, Keap1 del). Log-rank test. N = 100 flies per condition. (C) Keap1 del protected against Aβ42-induced sensitivity to paraquat, but did not add to the protective effect of lithium. Aβ42-expressing flies were more sensitive to toxicity induced by paraquat injection (*p*<0.001 comparing–RU to +RU). Lithium treatment alone significantly ameliorated sensitivity to paraquat (*p*<0.001 comparing +RU to +RU + LiCl 25mM). Reducing Keap1 alone protected against Aβ42 sensitivity to paraquat, but to a lesser extent than lithium alone (*p*<0.05 comparing +RU to +RU, Keap1 del and *p*<0.001 comparing +RU + LiCl 25 mM to +RU, Keap1 del) and did not enhance the protective effect of lithium (*p* = 0.402 comparing +RU + LiCl 25 mM to +RU +LiCl 25 mM, Keap1 del). No deaths were observed for mock injection flies for each condition. Log-rank test. N = 120–140 flies per condition.

As Nrf2 plays an important role in protecting against oxidative and xenobiotic stress, we investigated the nature of the Aβ42-induced molecular damage that was ameliorated by Keap1 inhibition (Fig 3). UAS-ArcAβ42>elavGS control and heterozygous Keap1 mutant flies were induced with RU486 for 14 days before measuring sensitivity to xenobiotic (DDT; dichlorodiphenyltrichloroethane) or oxidative (hyperoxia or paraquat) stressors (see methods).

Reducing Keap1 alone protected against Aβ42-induced sensitivity to DDT (Fig 3A) and paraquat (Fig 3C), but not hyperoxia (Fig 3B; +RU vs +RU, Keap1 del). Lithium treatment, on the other hand, protected against sensitivity of Aβ42 flies to DDT to a similar extent as Keap1 inhibition (Fig 3A), but more significantly protected against sensitivity to both hyperoxia (Fig 3B) and paraquat (Fig 3C)-induced oxidative damage. Moreover, when combined, lithium did not add significantly to the ability of reduced Keap1 to protect against the sensitivity of Aβ42 flies to DDT (Fig 3A, +RU, +LiCl 25 mM Keap1 del vs +RU, Keap1 del) and, conversely, reduced Keap1 did not add to the ability of lithium to protect against paraquat (Fig 3B) or hyperoxia (Fig 3C, +RU, +LiCl 25 mM Keap1 del vs +RU, +LiCl 25 mM). Consistent with the apparently different mechanisms of neuronal protection by Keap1 and lithium, this finding suggests that Keap1 inhibition acts mainly through protection against xenobiotic stress and lithium predominantly by limiting oxidative damage.

### Keap1 inhibition induces Aβ42 degradation

**Fig 4:**
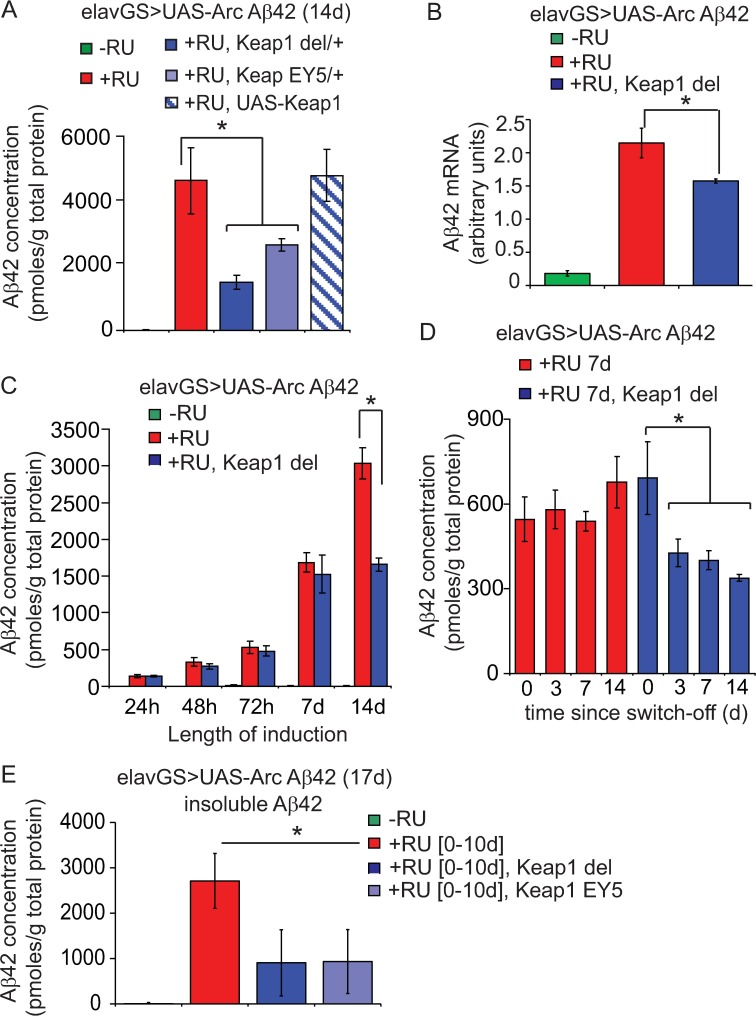
Keap1 inhibition enhances degradation of the Aβ42 peptide. (A) Lowering Keap1 reduced total Aβ42 peptide levels in 14 day-old flies. Data are presented as means ± SEM. *P*<0.05 comparing +RU or +RU, UAS-Keap1 to +RU, Keap1 del or +RU, Keap1 EY5 flies (one-way ANOVA and Tukey’s HSD). N = 4 biological repeats of 5 fly heads per sample. (B) Aβ42 mRNA in 14-day-old Keap1 del flies. Data are presented as means ± SEM. *P*<0.05 comparing +RU to +RU, Keap1 del flies (one-way ANOVA and Tukey’s HSD). N = 4 replicates of 20 flies per condition. (C) Reducing Keap1 lowered Aβ42 peptide levels, compared to controls. Data are presented as means ± SEM. *P*<0.05 comparing +RU to +RU, Keap1 del following 14 days of induction. No significant difference was observed between genotypes at all other time-points (two-way ANOVA and Tukey’s HSD). N = 3–4 replicates of 5 fly heads per condition. (D) Inhibition of Keap1 led to the degradation of Aβ42 peptide following removal of the inducer RU486. Data are presented as means ± SEM. *P*<0.05 comparing +RU 7d, Keap1 del flies at 0 to 3, 7 and 14 days following switch-off. No significant difference was observed between time-points following switch-off for +RU 7d control flies (two-way ANOVA and Tukey’s HSD). N = 4 repeats of 5 fly heads per condition. (E) Analysis of insoluble Aβ42 peptide (see methods) in 17-day-old Keap1 LOF flies following 10 days RU486 induction and 7 days switch-off. *P*<0.05 comparing +RU, to +RU, Keap1 del or +RU, Keap1 EY5 flies (one-way ANOVA and Tukey’s HSD). N = 3–4 biological repeats of 5 fly heads per sample.

In addition to increasing Nrf2/cncC activity, the protective effect of reducing Keap1 on Aβ42 toxicity correlated with a reduction in Aβ42 peptide levels (Fig 4A). Although Aβ42 mRNA was also slightly reduced by down-regulation of Keap1 (Fig 4B), the peptide levels were not altered until 14 days of age (Fig 4C), suggesting that reducing Keap1 may clear Aβ42 peptide by activating protein degradation mechanisms.

We have shown previously that Aβ42 peptide is stable for several weeks following induction in our inducible fly model[49]. To assess whether the peptide is indeed degraded as a consequence of reducing Keap1, we induced Aβ42 expression in Keap1^del^ and control flies for one week with RU486 then measured Aβ42 levels by ELISA at several time-points following transfer to non-RU486-containing medium (Fig 4D). Aβ42 levels were equivalent between heterozygous Keap1^del^ and control flies at the end of the induction period (0d since switch-off; age 7d), but then declined only in flies with reduced Keap1, starting from 3 days following switch-off (age 10d). This finding confirms that inhibition of Keap1 does indeed lead to degradation of the Aβ42 peptide. Moreover, analysis of insoluble proteins one week following RU486 induction for 10 days (Fig 4E), revealed that inhibition of Keap1 reduced the level of aggregated Aβ42.

**Fig 5:**
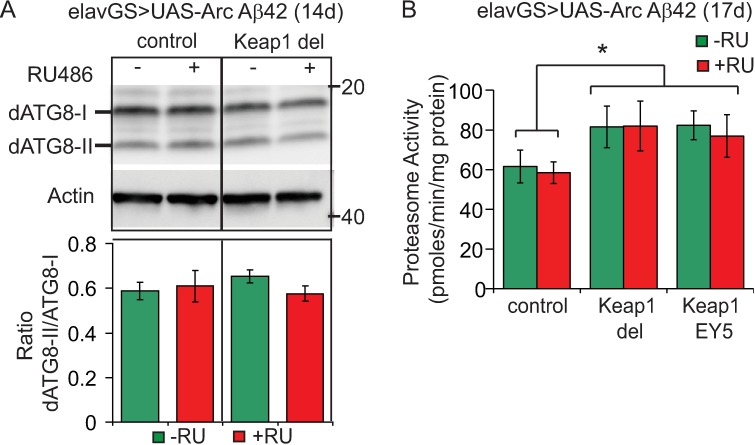
Keap1 inhibition and protein degradation pathways in Aβ42 flies. (A) Keap1 did not modify autophagy in Aβ42-expressing flies, as measured by the ratio of ATG8-II to ATG8-I levels. Data are presented as means ± SEM. *P*>0.05 (one-way ANOVA and Tukey’s post-hoc analysis). N = 4 biological repeats of 10 fly heads per condition. (B) Proteasome activity, as measured using the fluorogenic peptide substrate LLVY-AMC, was increased by Keap1 modification in Aβ42-expressing flies. Data are presented as mean activities (pmoles/min/mg protein) ± SEM. *P*<0.05 comparing–RU, +RU and–RU, Keap1 del to +RU, Keap1 del flies (one-way ANOVA and Tukey’s HSD). N = 4–5 repeats of 10 fly heads per condition.

Keap1-Nrf-2 signalling has been implicated in both autophagy and proteasomal degradation. p62, a selective autophagy substrate, competes with Nrf-2 for binding to Keap1[50][51] and Keap1 may activate autophagy directly by binding to p62[52]. However, we did not observe any alteration in autophagy activity upon reduction of Keap1 in the Aβ42-expressing flies, as measured by western blotting using an antibody specific for *Drosophila* ATG8 (Fig 5A). Proteasome subunits are transcriptional targets of Nrf2/cncC[53] and over-expression of cncC in flies, either directly or by reducing Keap1 levels, increases proteasome expression and activity[23]. Accordingly, we detected an increase in proteasome activity in the heads of Aβ42-expressing flies upon reduction of Keap1 (Fig 5B), at a time-point coinciding with reduced Aβ42 levels (age 14d). Pharmacological inhibition of this enhanced proteasome activity (S6A Fig), however, did not prevent the degradation of Aβ42 in response to Keap1 inhibition (S6B Fig), suggesting that this may not be the mechanism by which loss of Keap1 induces Aβ42 degradation. Further studies are therefore required to elucidate the precise clearance mechanisms mediating Aβ42 degradation following Keap1 inhibition.

### Direct Keap1-Nrf2 inhibitors protect against Aβ oligomer-induced synaptotoxicity in primary mouse neurons

Our data highlight Keap1 as a valid *in vivo* therapeutic target for AD. Current activators of Nrf2 are electrophilic compounds, which commonly act by modifying cysteine residues on Keap1 and thus disrupt its interaction with, and inhibition of, Nrf2[54]. Of these, the synthetic triterpenoids, such as Bardoxolone methyl (CDDO-Me), are potent Nrf2 activators and have exhibited therapeutic potential against several diseases, including neurodegeneration, in animal models and clinical trials[55]. One problem with the mechanism of action of these electrophilic Nrf2 activators, however, is the modification of cysteine residues on other targets[21,22], which may lead to side-effects in humans[22,55]. To improve specificity, compounds have more recently been designed to directly disrupt Keap1-Nrf2 binding, and these enhance Nrf2 activity *in vitro* [56] and in cells[22,57]. Activity of these compounds against disease models, however, remains to be examined.

**Fig 6:**
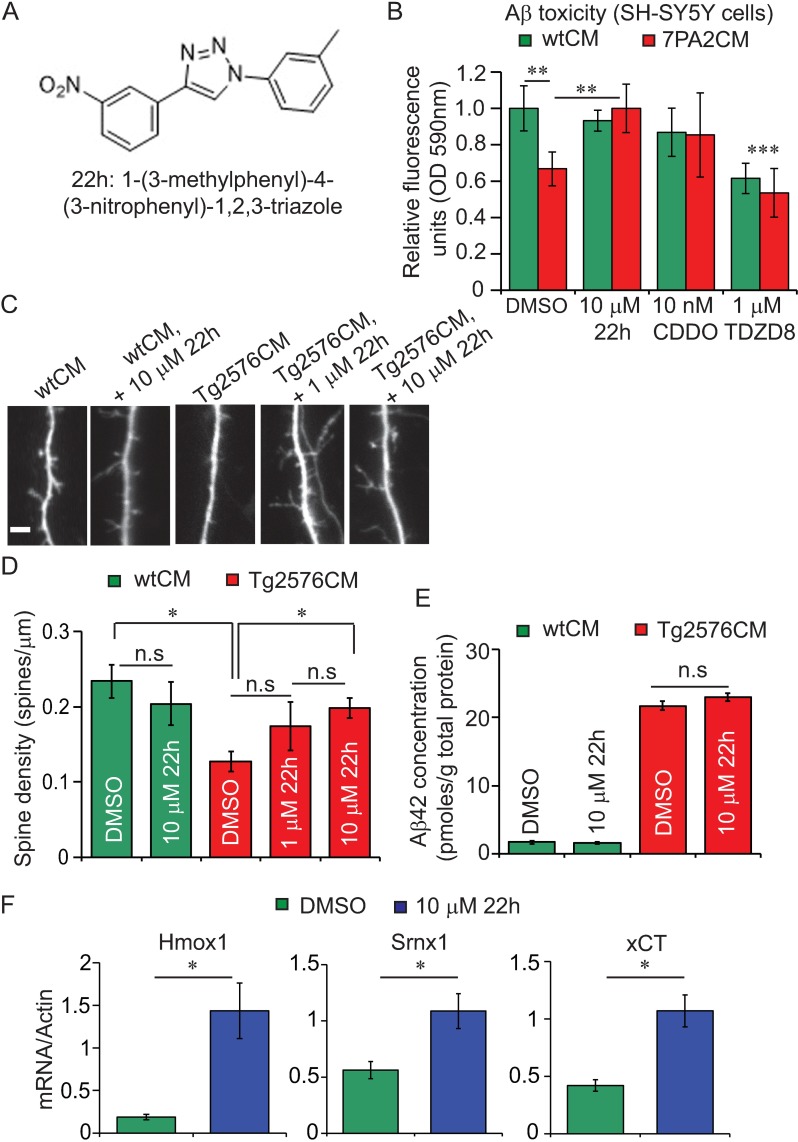
Direct Keap1-Nrf2 inhibitors protect mouse neurons from Aβ toxicity. (A) Molecular structure of compound 22h (1-(3-methylphenyl)-4-(3-nitrophenyl)-1,2,3-triazole[22]. (B) Aβ oligomer toxicity, as measured by sensitivity to resazurin (see methods), in SH-SY5Y cells treated with a 50% dilution of either WT or 7PA2 CHO cell conditioned medium (** *p*<0.01 comparing DMSO, wtCM to DMSO, 7PA2CM). An Nrf2 activator (CDDO-Me), the Keap1-Nrf2 disruptor (22h) and the GSK-3 inhibitor (TDZD8) were administered, at their EC_50_ concentrations for Nrf2 activation (see S7 Fig), and their effects on resazurin sensitivity measured in both WT and 7PA2-treated cells. At 1 μM, TDZD8 was toxic to cells under both treatment conditions (*** *p*<0.001 comparing TDZD8, wtCM and TDZD8, 7PA2CM to DMSO, wtCM). CDDO-Me and 22h exerted no toxicity at their effective concentrations in wtCM-treated cells (*P*>0.05). CDDO-Me slightly, but non-significantly, protected against Aβ oligomer toxicity (*P*>0.05 comparing DMSO, 7PA2CM to 10 nM CDDO, 7PA2CM cells) but 22h significantly protected against Aβ-induced damage (** *p*<0.01 comparing DMSO, 7PA2CM to 10 μM 22h, 7PA2CM). Data are presented as mean fluorescence units (as a ratio of DMSO, wtCM-treated cells) ± SEM and were analysed by two-way ANOVA and Tukey’s HSD post-hoc comparisons. N = 8 wells per condition. (C) Representative confocal images of mouse cortical neurons treated with Tg2576CM in the presence or absence of the Keap1-Nrf2 inhibitor, 22h. Scale bar 5μm. (D) Tg2576CM reduced total spine density of mouse cortical neurons (*p<0.05 comparing Tg2576CM, 0.01% DMSO to wtCM, 0.01% DMSO) and this was rescued by treatment with 22h in a dose-dependent manner (*p<0.05 comparing Tg2576CM, 0.01% DMSO to Tg2576CM, 10 μM 22h; *p*>0.05 comparing Tg2576CM, 1 μM 22h to Tg2576CM, 0.01% DMSO and Tg2576CM, 10 μM 22h). 22h did not exert cytotoxicity (*p*>0.05 comparing wtCM, 0.01% DMSO to wtCM, 10 μM 22h). Data are representative of means ± SEM and were analysed by one-way ANOVA and Tukey’s post-hoc analyses. N = 6–17 cells, from 2 independent wells, per condition. (E) Aβ42 peptide levels in conditioned media following 16h pre-treatment plus 24h CM treatment of WT mouse cortical neurons with or without 22h. (*p*>0.05 comparing 10 μM 22h to 0.01% DMSO for both wtCM and Tg2576CM). (F) mRNA expression of Nrf2 target genes, measured by qPCR, using extracts from primary cortical neurons from (E) above. Comparing 10 μM 22h to 0.01% DMSO, *p* = 0.008 (Hmox1), 0.013 (Srnx1) and 0.008 (xCT) (Wilcoxon rank sign test). N = 5–6 wells, per condition. 22h and DMSO data represent pooled means ± SEM from both wtCM and Tg2576CM-treated cells.

We tested the ability of a recently published direct Keap1-Nrf2 inhibitor, 22h (Fig 6A[22]), to protect against exogenous amyloid toxicity in cultured cells. At their EC_50_ concentrations for Nrf2 activation (S7 Fig), we first performed a comparative analysis of 22h, CDDO-Me and the GSK-3 inhibitor, TDZD-8, for protection against natural Aβ oligomer-induced toxicity[58] in SH-SY5Y cells (Fig 6B). Interestingly, Nrf2 and Keap1 protein levels were increased following 24 h Aβ oligomer treatment (S8 Fig), suggesting that the Keap1-Nrf2 pathway is indeed dysregulated by acute Aβ exposure. TDZD-8 was a poor activator of Nrf2, inducing its target gene NQO1 only at a single concentration of 1 μM (S7C Fig), and exerted toxicity in control SH-SY5Y cells (Fig 6B). Although CDDO-Me was a more potent activator of Nrf2 (S7D Fig), 22h significantly protected against Aβ toxicity, in comparison with both CDDO and TDZD-8, in this experimental paradigm (Fig 6B). This comparative analysis supports our suggestion that Keap1 may serve as a more efficient target, than inhibition of GSK-3, for the rescue of Nrf2-dependent effects of Aβ42 toxicity. Moreover, as TDZD-8 has previously been shown to protect against Aβ toxicity in primary neuronal cultures, our data suggest that the threshold for this protective effect of GSK-3 inhibition lies below that for its effects on Nrf2 activity. These findings indicate that direct Keap1-Nrf2 disruptors may protect against Aβ42 toxicity more effectively than established Nrf2 activators.

To further test the effects of Keap1-Nrf2 disruption on neuronal function in response to Aβ oligomers, we measured the effects of 22h in primary mouse neurons (Fig 6C). Conditioned medium obtained from Tg2576 mouse neurons (Tg2576CM) has previously been shown to contain oligomeric Aβ species at concentrations similar to those in human CSF, and to reduce spine density of GFP-transfected WT mouse cortical neurons[59]. We pre-treated GFP-transfected WT mouse neurons of 12 d *in vitro* (DIV), with 1 or 10 μM 22h for 16 h prior to administration of either wt or Tg2576 conditioned media (wtCM or Tg2576CM), with continued 22h treatment, for a further 24 h before examining spine density (see Materials & Methods). As previously reported[59], Tg2576CM reduced total spine density of cortical neurons compared to wtCM (Fig 6C and 6D). Strikingly, spine density was rescued by treatment with compound 22h (Tg2576CM, 0.01% DMSO vs Tg2576CM, 10 μM 22h) at non-toxic doses (wtCM, 0.01% DMSO vs wtCM, 10 μM 22h). Aβ42 levels in conditioned media were unchanged following treatment with 10 μM 22h (Fig 6E) which further correlated with increased expression of Nrf2 target genes[60] (Fig 6F).

These findings suggest that directly blocking the Keap1-Nrf2 interaction can protect neurons downstream of extracellular amyloid toxicity.

## Discussion

As the prevalence of ageing-related neurodegenerative diseases, such as Alzheimer’s, is predicted to rise dramatically[61], and with a lack of disease-modifying therapies currently available, there is an urgent need to find new therapeutic targets to slow down neuronal degeneration in these conditions. Accumulating evidence suggests that down-regulation of the cell protective transcription factor Nrf2 may enhance neuronal susceptibility to molecular damage [1, 62], and that its activation may confer neuronal protection[63]. Nrf2 is, therefore, a promising novel target for the prevention of several neurodegenerative diseases, but its activation must be tightly regulated to have beneficial effects[23,24]. Moreover, although current activators of Nrf2, including electrophilic compounds, such as sulforaphane, and the more potent bardoxolone methyl (CDDO-Me), can exert neuroprotective effects[20,64], all of these compounds are indirect activators and likely to exert multiple off-target effects[55], potentially leading to toxicity. A better understanding of the precise mechanisms regulating Nrf2 activity (S1 Fig) under particular pathological conditions would, therefore, enable the design of drugs targeted to the prevention of specific diseases with minimal side-effects. Our work aimed to investigate the mechanisms regulating Nrf2 inhibition specifically in response to the AD-related Aβ42 peptide, with a view to identifying novel targets for the prevention of AD and other ageing-related neurodegenerative conditions.

### Nrf2 activity in Alzheimer’s disease

**Fig 7:**
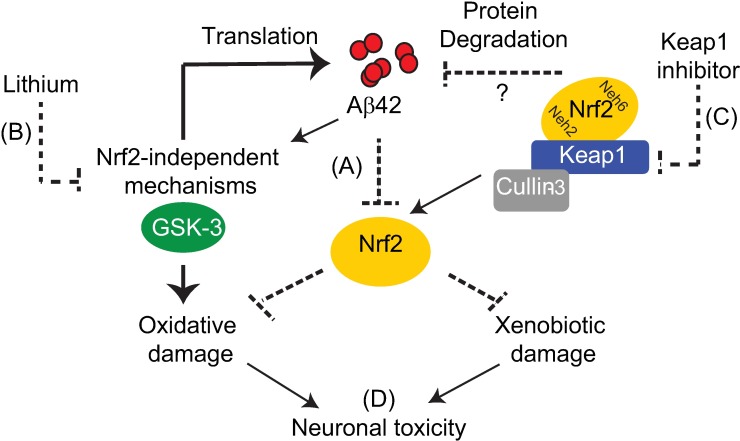
Keap1 and GSK-3 in the regulation of Nrf2 in Alzheimer’s disease. (A) Aβ42 peptide inhibits activity of Nrf2, and this may explain the increased presence of xenobiotic and oxidative stress markers observed in Alzheimer’s disease. (B) Although lithium can activate Nrf2 at high concentrations, its protective effect against Aβ42 toxicity appears to be mainly Nrf2-independent, reducing Aβ42 levels by inhibiting translation[47] and preventing oxidative damage. More specific GSK-3 inhibitors are required to confirm the precise role of GSK-3 in rescuing Nrf2 deficits in neurodegenerative disease. (C) Genetic and pharmacological inhibition of Keap1 can rescue Aβ42-induced Nrf2 inhibition and neuronal toxicity by preventing xenobiotic damage and activating degradation of Aβ42 peptide. (D) Keap1 inhibitors may serve as effective therapies for AD and, in combination with GSK-3 inhibitors, may provide added benefits in preventing neurodegeneration through non-overlapping mechanisms.

Our study firstly confirms, in *Drosophila*, previous reports that Nrf2 target genes are down-regulated in AD brain[11], and in mouse models of AD[13,65,66]. Some studies suggest that this inhibition may be mediated by Aβ42 directly, with exogenous, synthetic, Aβ42 peptide reducing Nrf2 target gene expression in primary mouse neurons[13], and blocking Nrf2 nuclear translocation following injection into the hippocampus of rat[65] and mouse[66] brain. As Aβ42 peptide is expressed in our fly model independently of APP processing, our study further confirms that this direct effect on Nrf2 activity occurs in response to naturally-derived Aβ42 peptide conformations *in vivo* (Fig 7B). Importantly, other disease-related proteins, including tau and C9orf72 DPRs, also suppressed Nrf2/cncC signalling, but the non-toxic Aβ40 peptide did not. This suggests that inhibition of the Nrf2 pathway is a generalised response to the accumulation of aberrant proteotoxic proteins.

The mechanisms mediating the effect of Aβ42 on Nrf2 activity remains to be established. Although a recent report, using sweAPP-expressing cells, has suggested that Nrf2 transcripts can be replenished by altering DNA methylation [67], Aβ42 did not alter mRNA expression of Nrf2/cncC, or of its co-transcription factor MafS, in our *Drosophila* model. This suggests that *in vivo* Nrf2 de-regulation in AD may be post-transcriptional. We have shown that Aβ42 oligomers increased Nrf2 and Keap1 proteins in SH-SY5Y cells after 24 h treatment, suggesting that Nrf2 is initially stabilised in response to acute amyloid exposure. This may represent a protective response to the initial toxic insult, as similar effects on Nrf2 have been observed at early time-points following transient focal ischaemia and correlate with preservation of peri-infarct regions of the brain under these conditions [68]. Keap1 is also an Nrf2 target gene [35], however, and may subsequently be upregulated to control Nrf2 activity following the initial exposure to Aβ42 in our study. If sustained, this increase in Keap1 levels could provide a potential mechanism for the inhibition of Nrf2 observed in AD brain and other chronic neurodegenerative conditions [11]. This hypothesis is supported by observations that Nrf2 protein is downregulated and Keap1 upregulated in mouse brain following 15 days exposure to synthetic Aβ42 [66]. Further work is required, however, to investigate the detailed timing of these events following chronic *in vivo* exposure to natural Aβ oligomers.

### GSK-3 as a target for AD: Nrf2-independent mechanisms?

Since Aβ42 did not directly affect Nrf2/cncC gene expression, we investigated the role of its upstream inhibitors, GSK-3 and Keap1, on toxicity. GSK-3 has a well-documented role in Alzheimer’s, and is suggested to provide a pivotal connection between the characteristic amyloid plaque and neurofibrillary tangle pathologies of this disease[26]. Activation of GSK-3 has been proposed to exert neuronal toxicity by many mechanisms, including increasing apoptosis and inflammation, and impairing axonal transport, synaptic function, cell cycle regulation and adult neurogenesis [26]. Conversely, several GSK-3 inhibitors, including lithium, protect against AD-pathology in mice[69–71] and some have been tested in clinical trials for AD[72]. Although GSK-3 inhibition increases Nrf2 activity in AD models[73], however, only one study has shown epistatically that Nrf2 mediates the neuroprotective effect of lithium treatment against paraquat toxicity in cells[74]. The role of Nrf2 in mediating the protective effect of GSK-3 inhibition in AD has also not been empirically investigated.

Our current study confirms that lithium treatment increases transcription of Nrf2 target genes in a dose-dependent manner. Concentrations of lithium required to activate Nrf2 (≥50 mM-100 mM LiCl), however, have previously been shown to exert toxicity in *Drosophila*[42,47]. Lower doses of lithium (25 mM) were sufficient to protect against Aβ42 toxicity, to a level comparable with reducing Keap1, but did not significantly activate Nrf2/cncC. Moreover, genetically reducing Nrf2/cncC function did not prevent the lifespan-extending effects of lithium, in Aβ42 flies, even at a maximizing concentration (50 mM). Our data, therefore, suggest that lithium mediates neuroprotection against Aβ42 independently of its effects on Nrf2 (Fig 7B). Consistent with our previous observations that lithium (10–100 mM) reduces Aβ42 peptide levels by inhibiting translation[47], early lithium administration reduced Aβ42 peptide, from the point of induction, in our current study and this was required for lithium to exert its protective effects.

These findings are further supported by our observation that the specific GSK-3 inhibitor TDZD-8 has a narrow window for Nrf2 activation in cultured cells, inducing its target gene NQO1 only at a single concentration of 1 μM (Fig 6B). At concentrations ≤ 1 μM TDZD-8 has been described to prevent Aβ-induced reductions in spine density in mouse neurons[59], but doses ≥ 1 μM exerted toxicity (Fig 6B and [59]). Together these data suggest that the threshold for protection against Aβ toxicity by GSK-3 inhibitors lies below the concentration required for activation of Nrf2 in neuron. This supports the hypothesis that blocking GSK-3 exerts its protective effects independently of Nrf2.

### Keap1 as a target for AD: Nrf2-dependent mechanisms?

Contrary to our findings with lithium, we provide the first *in vivo* evidence that Keap1 inhibition can exert neuroprotective effects in response to Aβ42 by ameliorating deficits in Nrf2 activity (Fig 7C). As with *Drosophila* models of PD[32,33], heterozygous loss of Keap1 protected against Aβ42-induced lifespan-shortening and climbing defects in the fly. Moreover, we show that this protective effect of reducing Keap1 correlates significantly with a rescue of Nrf2/cncC activity in response to Aβ42.

Only one previous study has addressed the role of Keap1 inhibition, specifically, in AD. Keap1 RNA interference increased expression of Nrf2 target genes, protected against synthetic Aβ42-induced cytotoxicity and oxidative damage to proteins and lipids, and enhanced autophagy in cultured cells[30]. Our study adds to these *in vitro* findings, demonstrating that Keap1 inhibition rescues Aβ42-induced Nrf2 deficits, and protects against neuronal decline *in vivo*. These effects occur in correlation with prevention of xenobiotic and, to a lesser extent, oxidative damage as well as the degradation of endogenous, aggregated, Aβ42 peptide.

Using primary cortical neurons, our findings further confirm that direct pharmacological inhibition of Keap1-Nrf2 binding can protect against neuronal damage downstream of extracellular Aβ42 oligomers. The mechanisms by which reducing Keap1 enhances degradation of Aβ *in vivo*, however, requires further investigation. Autophagy, as measured by cleavage of ATG8, was unaltered in response to Keap1 inhibition in our Aβ42 expressing flies. Consistent with the established effect of Nrf2 on transcription of proteasome subunits[23], loss of Keap1 enhanced proteasome activity in Aβ42 flies, but blocking this increase, using the proteasome inhibitor Bortezomib, did not prevent the reduction in Aβ42 levels. This suggests that the reduction of aggregated Aβ42 in response to Keap1 inhibition may not be directly mediated via enhanced autophagy or proteasomal degradation. Nrf2 has also been implicated to play a role in the unfolded protein response (UPR), serving as a target for PERK[75] and activating transcription of several components of the ER associated degradation (ERAD) pathway[76], including chaperones and ubiquitin-conjugating enzymes, in addition to proteasome subunits and autophagy. Future studies will therefore be required to investigate the functional role of such protein quality control pathways, potentially upstream of the proteasome and autophagy, in the clearance of Aβ42 following Keap1 inhibition *in vivo*.

Overall, our data point to Keap1, in comparison with GSK-3, as an efficient target for Nrf2 activation in response to Aβ42 toxicity *in vivo*. Recent advances in the development of direct inhibitors of the Keap1-Nrf2 binding domain may, therefore, enable the prevention of Nrf2 deficits in neurodegenerative diseases with minimal side-effects. Our study shows for the first time that a direct, small molecule inhibitor of Keap1-Nrf2 binding, 22h[22], can ameliorate synaptotoxicity in response to naturally-derived Aβ oligomers in mouse cortical neurons. As synaptic loss correlates well with cognitive decline[77], our work suggests that these compounds show promise as therapeutic agents for AD. Moreover, as this is the first demonstration that these compounds can prevent neuronal toxicity, our findings warrant their investigation in other neurodegenerative conditions.

### Combined benefits of Keap1 and GSK-3 inhibition for AD?

Both GSK-3 inhibitors[71] and Nrf2 activators[55] can exert toxicity due to off-target effects and thus it is important that modifiers of these pathways achieve therapeutic benefits at low doses. More specific inhibitors of each of these targets are currently being developed[22,71]. It has also been postulated that combined inhibition of both GSK-3 and Keap1 may activate Nrf2, and confer protection, at lower doses than either intervention alone, hence limiting their detrimental effects[25].

We present the first *in vivo* study showing that combined Keap1 and GSK-3 inhibition, by lithium, confers additive protective effects against Aβ42 toxicity. Keap1 deletion and lithium treatment extended lifespan and improved climbing ability of Aβ42-expressing flies to a greater extent than either manipulation alone. Additionally, reducing Keap1 limits the dose of lithium required to reach maximal levels of protection since heterozygous Keap1^del^ combined with low dose (25 mM) lithium extended lifespan of Aβ42 flies to a similar extent as high dose (50 mM) lithium alone. Our data, however, do not predict that these additive protective effects are due to mechanisms converging on Nrf2. Rather, low dose lithium treatment exerts protective effects independently of Nrf2 activation, whereas the neuroprotective effects of Keap1 inhibition correlate strongly with increasing Nrf2. Hence, the additive nature of combined lithium and Keap1 inhibition appears to be mediated through divergent, complementary protective mechanisms.

Oxidative[2,3] and xenobiotic damage[9] are key features of AD brain and may potentially be explained by the down-regulation of Nrf2, which is also observed in several neurodegenerative diseases. Consistent with this idea, Keap1 inhibition and, therefore, Nrf2/cncC activation, correlated strongly with protection against Aβ42-induced sensitivity to the xenobiotic DDT in our fly model. Conversely, Keap1 inhibition exerted minimal protection against oxidative damage in comparison with lithium treatment, which strongly protected against Aβ42-induced sensitivity to both paraquat and hyperoxia-induced damage. As therapeutic concentrations of lithium, which can prevent oxidative damage, did not strongly activate Nrf2/cncC in our flies, this suggests that rescue of Nrf2 activity is not required to protect against oxidative damage in response to Aβ42. Although pharmacological activation of Nrf2, has been shown to protect against oxidative damage, in response to Aβ42 peptide, in cells[78,79] and in animal models of AD[65,66], conflicting reports have also described protective effects against such damage that are mediated independently of Nrf2[80]. Moreover, studies showing protection against Aβ42-induced oxidative damage in response to GSK-3 inhibition, using antisense oligonucleotides[73] and in response to lithium[81], have not demonstrated a causal role of increasing Nrf2 activity. Our direct comparison of Keap1 and GSK-3 pathways, however, reveals that Nrf2 activity and prevention of Aβ42-induced oxidative damage do not strongly correlate.

Finally, combining lithium treatment with manipulation of Keap1 did not confer additional protection against oxidative and xenobiotic damage respectively. Overall this suggests that Keap1 and lithium treatment combine to maximise protection against AD-phenotypes, through divergent effects on Nrf2 and by additively protecting against Aβ42-induced oxidative and xenobiotic damage (Fig 7D).

## Conclusions

Our findings provide compelling support for the use of direct Keap1-Nrf2 inhibitors for the treatment of neurodegenerative diseases, particularly AD. Future work is warranted to develop these compounds further for *in vivo* use, and to investigate their effects in combination with other established therapeutic targets for AD, such as specific GSK-3 inhibitors.

## Materials and methods

### Fly stocks and husbandry

Stocks were maintained at 25°C on a 12:12-h light:dark cycle at constant humidity on a standard sugar-yeast (SY) medium (15gl^-1^ agar, 50 gl^-1^ sugar, 100 gl^-1^ autolysed yeast, 100gl^-1^ nipagin and 3ml l^-1^ propionic acid). Adult-onset neuronal-specific expression of Arctic mutant Aβ42 peptide was achieved by using the elav GeneSwitch (elavGS)-UAS system (GAL4-dependant upstream activator sequence) and treatment with 200 μM mifepristone (RU486), as previously described[34]. ElavGS was derived from the original elavGS 301.2 line and obtained as a generous gift from Dr H. Tricoire (CNRS, France). Aβ lines used in Fig 1 were: UAS-attP Aβ lines, as previously published [36], and UAS-WT Aβ42x2, obtained from Prof. P. Fernandez-Funez (University of Florida, USA)[37]. Random insertion UAS-ArcAβ42 was obtained from Dr D. Crowther (University of Cambridge, UK) and was used in all other experiments. UAS-0N3R tau line was obtained from Guy Tear (Kings College London, UK). UAS-C9orf72 (GR)100 flies are published[40]. Keap1^del^ was generated by P-element mediated male recombination using the P-element insertion line, Keap1[EY02632][42], Keap1^EY5^ and *gstD1*-GFP lines were obtained from Dr D. Bohmann (University of Rochester, USA), and cncCK6 mutant flies, originally generated in the laboratory of William McGinnis (University of California, San Diego, USA), from Dr A. Whitworth (University of Cambridge, UK). All fly lines were backcrossed six times into the w^1118^ genetic background.

### Lithium treatment

LiCl (Sigma) was dissolved in ddH_2_0 at a concentration of 5 M before diluting to the indicated final concentrations in SYA medium.

### Bortezomib treatment

Bortezomib (New England Biolabs) was dissolved in ethanol at a stock concentration of 10 mM, and stored frozen at– 20°C, before diluting to the indicated final concentrations in SYA medium.

### Lifespan analyses

Flies were raised at a standard density on SY medium in 200 mL bottles. Two days after eclosion once-mated females were transferred to experimental vials containing SY medium with or without RU486 at a density of 10 flies per vial. Deaths were scored and flies were transferred to fresh food 3 times per week. Data are presented as cumulative survival curves, and survival rates were compared using log-rank tests.

### Negative geotaxis

Climbing assays were performed using methods adapted from Sofola O et al., 2010 [34]. Briefly, 15 adult flies were placed in a vertical glass column (SciLabware), tapped to the bottom, and their ability to climb subsequently analysed. Flies reaching the top (above 10 cm) and flies remaining at the bottom (below 3 cm) of the column after a 30 sec period were counted. Scores recorded, from three trials per biological repeat, were the mean number of flies at the top (n_top_), the mean number of flies at the bottom (n_bottom_) and the total number of flies assessed (n_tot_). A performance index (PI) defined as ½ (n_tot_ + n_top_—n_bottom)/_ n_tot_) was calculated. Data are presented as the mean PI ± SEM obtained in three independent repeats for each group.

### cncC activity

cncC activity was measured by crossing UAS-ArcAβ42;elavGS flies to *gstD1*-GFP reporter flies, expressing green fluorescent protein (GFP) under the control of a 2.7kb genomic sequence upstream of the cncC target gene *gstD1* [35], and analyzing GFP levels by western blotting.

### Western blotting

Fly heads were homogenized in 2× laemmli sample buffer containing 100mM DTT. Proteins were separated by SDS-PAGE at 150V for 1h using 10% Bis-Tris gels and Mes-SDS running buffer (Invitrogen). Gels were then transferred to nitrocellulose membrane, incubated in a blocking solution containing 5% milk proteins in TBST for 1h at room temperature, then probed with GFP (Cell Signaling, 2955S, 1:1000), ATG8 (custom made anti-rabbit polyclonal against peptide EP113385 (Eurogentec) [42], 1:1000,) or actin (Santa Cruz, sc-47778, 1:5,000) primary antibodies overnight at 4°C. Anti-horseradish peroxidase (HRP)-conjugated secondary antibodies (Abcam, 1:12,000) were used and blots were developed using the enhanced chemiluminescence method (ECL) according to the manufacturers’ instructions (Luminata Crescendo; Millipore). Proteins were visualized using a luminescent image analyzer (ImageQuant LAS 4000; GE Healthcare) and relative intensities measured using ImageQuant software. Proteins of interest were expressed as a ratio relative to actin levels in each sample.

### Quantitative RT-PCR

RNA extraction, cDNA synthesis and quantitative PCR (qPCR) reactions were performed as previously published[34,49]. For gene expression in *Drosophila*, primers, 5’ to 3’, were as follow: Aβ forward GATCCTTCTCCTGCTAACC and reverse CACCATCAAGCCAATAATCG; cncC forward GAGGTGGAAATCGGAGATGA and reverse CTGCTTGTAGAGCACCTCAGC; MafS forward AGATCGTTCGGATGAAGCAG and reverse GTCTCCAGCTCGTCCTTCTG; gstD2 forward CATCGCCGTCTATCTGGTGGA and reverse GGCATTGTCGTACCACCTGG; and eIF-1A reference gene forward ATCAGCTCCGAGGATGACGC and reverse GCCGAGACAGACGTTCCAGA[82]. Genes of interest were expressed as a ratio relative to eIF-1A.

For gene expression in primary mouse neuronal cultures, primers 5’ to 3’ were: Hmox1 forward AGCACAGGGTGACAGAAGAG and reverse GGAGCGGTGTCTGGGATG, Srnx1 forward GACGTCCTCTGGATCAAAG and reverse GCAGGAATGGTCTCTCTCTG, and xCT forward ATACTCCAGAACACGGGCAG and reverse AGTTCCACCCAGACTCGAAC, as previously published [60]. Reference gene primers were to mouse actin forward AACCGTGAAAAGATGACCCAGA and reverse CACAGCCTGGATGGCTACGTA. Genes of interest were expressed as a ratio relative to actin.

### Quantification of total and insoluble Aβ42 peptide

Total Aβ42 peptide, from fly heads, was extracted into guanidinium HCl (GnHCl) buffer based as previously described[34,83]. Alternatively, soluble and insoluble Aβ pools were isolated by differential centrifugation followed by formic acid extraction, as previously described [49]. Aβ42 levels were then measured using the High Sensitivity Human Amyloid Aβ42 ELISA kit (Millipore). Samples were diluted 1:100, for total Aβ42, or 1:10, for insoluble Aβ42, in dilution buffer and ELISA performed according to the manufacturers’ instructions. Protein extracts were quantified using the Bradford protein assay (Bio-Rad laboratories Ltd, UK) and the amount of Aβ42 in each sample expressed as a ratio of the total protein content (pmoles/g total protein).

For cell culture, conditioned media was removed from cells, following compound treatment, and diluted 1:2 in dilution buffer before measurement of Aβ42 levels by ELISA as described above.

### Proteasome activity

Fly heads were homogenized in 25 mM Tris, pH 7.5 and protein content determined by Bradford assay. Chymotrypsin-like peptidase activity of the proteasome was assayed using the fluorogenic peptide substrate Succinyl-Leu-Leu-Val-Tyr-amidomethylcoumarin (LLVY-AMC), as previously described[49]. Proteasome activity was determined as the slope of AMC accumulation over time per mg of total protein (pmoles/min/mg).

### Stress assays

Flies were prepared as for lifespan experiments then aged to the indicated time-points before measuring stress resistance. For xenobiotic stress, flies were exposed to DDT vapour, 1 mg/mL diluted in acetone, in glass vials in the absence of food. For oxidative stress, flies were subjected to hyperoxia (95% oxygen; PROOX model 110, Biospherix, USA) or injected with paraquat (75 nLs of 1 mg/ml in Ringers buffer) and maintained on SYA media containing RU486 ± LiCl as indicated. As survival times were short in these experiments flies were not transferred to fresh food. Survival under each stress condition was then monitored by recording the number of deaths every 2 hours from the start of decline. Data are presented as cumulative survival curves, and survival rates were compared using log-rank tests.

### Microarray analyses

The raw microarray data generated in this study are deposited in ArrayExpress (http://www.ebi.ac.uk/arrayexpress) with identifier E-MTAB-4611. UAS-ArcAβ42/+;elavGS/+ flies were treated, for 17 days, with standard SY medium alone (-RU) or medium containing RU486 in the absence (+RU) or presence of 100 mM Lithium Chloride (+RU, +LiCl). Flies used for microarray analyses were snap-frozen in liquid nitrogen and, for each array, RNA extracted from 200 heads using RLT buffer + 0.01% β-mercaptoethanol and purified with RNeasy columns (Qiagen, Valencia, CA, USA) following the manufacturer's instructions. The quality and concentration of RNA was confirmed using an Agilent Bioanalyzer 2100 (Agilent Technologies, Santa Clara, CA, USA), and further procedures followed the standard Affymetrix protocol. All samples were hybridized to the *Drosophila* Genome 2.0 Genechip. In total, 4–5 biological replicates of each condition (-RU, +RU and +RU, +LiCl) were performed.

### Differential expression analysis

Raw data (cel files) were processed to correct for probe-sequence biases, by using bioconductor's package gcrma (http://www.bioconductor.org) in R (http://www.r-project.org). Affymetrix's MicroArray Suite 5.0 (bioconductor's package affy) was used to determine present target transcripts[84]. Transcripts were deemed present if the *p*-value was <0.111 and absent otherwise. The raw data were summarized and normalized by using Robust Multichip Average (rma function, part of bioconductor's package affy [85]. In order to identify differentially expressed genes a linear model was fitted and differential expression was assessed using the empirical Bayes moderated *t*-statistic as implemented in R's limma package [86]. *P*-values were adjusted for multiple hypothesis testing by applying the Benjamini and Hochberg correction for false discovery rate. Summarized probe-sets were mapped to transcripts using R's package "drosophila2.db". Transcripts not mapping to any known or predicted genes were excluded from further analysis. The following freely available gene expression microarray datasets were used: control vs. *hsp70*-CncC (E-GEOD-30087).

### Gene Ontology analysis

The Wilcoxon rank sum test, as implemented in Catmap[87], was used to perform functional analysis, that is significant enrichment of Gene Ontology categories. FlyBase (http://flybase.org) gene identifiers were mapped to Gene Ontology identifiers (FlyBase version FB2014_01). Ranks of genes were based on the *p*-value derived from the Bayes *t*-statistic for differential expression. To account for multiple hypothesis testing, an enrichment of GO terms was deemed statistically significant if the *p*-value derived from the wilcoxon rank sum test was ≤1.0x10^-05^. Gene lists were sorted by log-fold change and *p*-value. For all microarray experiments two sets of lists were derived; a gene list comprising most differentially up-regulated (log-fold change > 0) genes at the top of the list and most differentially down-regulated genes (log-fold change < 0) at the bottom of the list (termed up-to-down) and *vice versa* (termed down-to-up). If a GO category was found to be statistically significant in the up-to-down list, this GO was referred to as up-regulated, meaning that a large enough proportion of the genes in this GO category were found to be up-regulated or at the top of the list. If a GO category was found to be statistically significant in the down-to-up list, this GO was referred to as down-regulated, meaning that a large enough proportion of the genes in this GO category were found to be down-regulated or at the top of the list.

### Statistical significance of Gene Ontology (GO) sets

Statistical significance of overlaps of GOs in two microarray experiments was determined using fisher's exact test. To account for multiple hypothesis testing a *p*-value cut-off of ≤1.0x10^-05^ was used.

### SH-SY5Y neuroblastoma cell culture

SH-SY5Y cells were incubated in a humidified atmosphere at 37°C, 5% CO_2_ in Dulbecco’s modified medium (DMEM) supplemented with 4.5g/L glucose, 10% FBS, 1% penicillin- streptomycin, and differentiated with 10 μM retinoic acid. Cells were seeded at an appropriate density in 96-well plates prior to subsequent analyses.

### NQO1 induction assay

SH-SY5Y cells were seeded at a density of 2 x 10^4^ cells per well in a 96-well plate and cultured for 2 days before treatment for 24 hours with compound or vehicle (0.1% DMSO final concentration). Cells were then lysed in 50 μl/well lysis buffer (0.1% Tween20 in 2 mM EDTA [pH 7.5]) for 15 mins at room temperature. 200 μl enzyme reaction mixture (25 mM Tris buffer [pH 7.5] containing BSA [0.067%], Tween20 (0.01%), FAD (5 μM), Glucose 6 Phosphate (G6P) (1 mM), NADP (30 μM), G6P dehydrogenase (40 units), MTT (0.03%), and menadione (50 μM)) was added to each well. After 5 min at room temperature, 40 μl/well stop solution (10% SDS) was added and the absorbance at 595 nm measured. The background optical density was measured using wells containing lysis buffer, enzyme and stop solutions without SH-SY5Y cells. The optical density values at 595 nm were averaged and the background corrected ratio of optical densities (compound treated/control) was calculated.

### Resazurin toxicity assay

SH-SY5Y cells were seeded at a density of 2 x 10^4^ cells per well in a 96-well plate. Cells were pre-treated with compound or vehicle overnight before either Aβ-conditioned Chinese Hamster Ovary (CHO) cell media (7PA2CM) or WT-conditioned CHO media (wtCM) was added to wells at a dilution of 50% for 24 hours. Resazurin (final concentration 20 μM) was added to wells and incubated for 4 hours at 37°C, 5% CO_2_. The resulting fluorescence intensity was measured at 590 nm. The relative fluorescence values were averaged and normalised to wtCM, DMSO-treated control intensities.

### Immunostaining

SH-SY5Y cells were seeded at a density of 6,000 cells per well, in a 96-well plate then, after 24 hours, treated with 50% 7PA2CM or wtCM for a further 24 hours. Plates were fixed with 4% PFA (v/v) for 15 minutes, washed three times with PBS, permeabilized with 0.5% Triton-X before staining with primary antibodies against Keap1 (1:50; ab150654, Abcam) and Nrf2 (1:100; ab62352, Abcam) in blocking solution containing 10% goat serum and 3% BSA overnight at 4 C. After 3 washes in PBS secondary antibodies (α rabbit Alexa 594, ab150080, α mouse FITC, ab6785, Abcam) were applied. Nuclei were stained in a PBS solution of 10 μM Hoechst 33342 before high-resolution digital imaging using a Zeiss LSM700 confocal microscope.

For quantification of Nrf2 and Keap1 staining, and cell morphology, 250 cells per well were imaged using the IN Cell Analyzer 6000 automated laser-based imaging platform with confocal modality (GE Healthcare) at 40X magnification. Automated image analysis was conducted with the IN Cell Developer software using custom-developed analysis protocols. Cell nuclei were identified by acquiring images in the DAPI channel (405 nm excitation, 455/50 emission). Whole cells were identified using images of Keap1 from the FITC channel (488 nm excitation, 524/48 emission). Images were also acquired of Nrf2 in the dsRed channel (561 nm excitation, 605/52 nm emission). The whole-cell intensity of Keap1 FITC and Nrf2 Alexa 594 was measured and expressed as average fluorescence units per cell for each well. N = 5 wells per condition.

### Preparation of Aβ-oligomers from Tg2576 mouse neuron conditioned media

To obtain transgenic conditioned medium (TgCM) enriched in Aβ, neurons from Tg2576 mice were maintained for 14 days *in vitro* (DIV) without changing the medium[88]. Medium from wild type neurons from littermates, wild type conditioned medium (wtCM), was used as a control. The genotype of the animals was determined by polymerase chain reaction on DNA obtained from fibroblasts.

### Culture and treatment of primary mouse cortical neurons

Wild type primary neurons were obtained from cerebral cortex of CD1 mouse embryos, at embryonic day 15, as previously described [89]. Neurons were plated to a density 6×10^5^ viable cells/35-mm^2^ on glass-bottomed dishes (MatTek Corporation, Ashland, MA, USA) previously coated with poly-D-lysine (10 μg/ml) for at least 1 h at 37°C. Cultures were maintained at 37°C with 5% CO_2_, supplemented with neurobasal medium with 2% B27 nutrient, 2 mM L-glutamine, penicillin (100 units/ml) and streptomycin (100 μg/ml). At 12 days *in vitro* (DIV) neurons, transfected on day 7 with the plasmid peGFP-N1 (Clontech, Mountain View, CA) using lipofectamine 2000 (Invitrogen), were pre-treated with the Keap1 inhibitor, 22h (10 μM[22]), or 0.1% DMSO, for 16 h. Conditioned medium (CM) from Tg2576 transgenic, Aβ-enriched, or wild type mouse neurons, with and without 22h, was then added for a further 24h before analysis.

### Analysis of neuronal morphology

Neuronal morphology was assessed at 14 DIV by high-resolution digital imaging using a Zeiss LSM700 confocal microscope and analysis using NeuronStudio software (CNIC, Mount Sinai School of Medicine). Spine density was defined as the number of spines per micrometer of dendrite length according to previously published protocols[59]. Dendritic spine densities were calculated from 6–17 neurons per condition.

### Statistical analyses

Data are presented as means ± SEM obtained in at least three independent biological samples. Log-rank, analysis of variances (ANOVA) and Tukey’s HSD (honestly significant difference) post-hoc analyses were performed using JMP (version 11.0) software (SAS Institute, Cary, NC, USA).

### Ethics statement

Animals were maintained and treated in accordance with the Animals (Scientific Procedures) Act, 1986, following approval by the Animal Welfare and Ethical Review Body (AWERB), KCL, and the Home Office Inspectorate, and performed in accordance with the European Communities Council Directive of 24 November 1986 (86/609/EEC).

## Supporting information

S1 Fig Dual degradation model of Nrf2 regulation. Under basal conditions (A) Keap1 binds to, and sequesters, Nrf-2 in the cytoplasm and actively targets it for ubiquitination by a Cullin3-based E3 ligase complex, thus enabling its degradation by the proteasome[1]. During conditions of oxidative or xenobiotic damage (B), Keap1 is inhibited by reactive oxidant species (ROS) and electrophiles, thus facilitating the stabilisation of Nrf-2 and enabling its translocation to the nucleus. Nrf-2 then forms dimers with small Maf (musculo-aponeurotic fibrosarcoma oncogene) proteins, which subsequently bind to and activate transcription of antioxidant response element (ARE)–containing cell protective genes. Alternatively, GSK-3 can inhibit Nrf-2 (C) by targeting it for ubiquitination by a β-TrcP-Cullin1 complex[43], through mechanisms that are independent of Keap1. A dual-degradation model for regulation of Nrf-2 under different pathophysiological conditions has been hypothesized[25].
(TIF)

S2 Fig Aβ42 inhibits expression of cncC target genes in heads, but not bodies. (A) cncC activity was measured in 14-day-old Arc Aβ42-expressing fly heads and bodies. ** p<0.01 comparing +RU to–RU in heads. For bodies only, no significant effect of ArcAβ42 on *gstD1*(ARE)–GFP reporter expression was observed compared to controls (*p*>0.05 comparing +RU to–RU controls, student’s t-test). Data represent means ± SEM. N = 4 replicates of 10 flies per condition.
(B) Venn diagrams showing the overlap of GO categories differentially altered by Aβ42 and cncC[38], most of which were reciprocally regulated. Few GO terms were significantly up-regulated by both Aβ42 and cncC activation, and no significant overlap was observed between genes down-regulated in both conditions.
(TIF)

S3 Fig Nrf2/cncC activity in response to disease-related proteotoxic proteins. (A) Western blot analysis of *gstD1*(ARE)–GFP reporter expression in w1118 controls and flies over-expressing human 0N3R tau or C9orf72 (GR)100 DPRs in adult neurons. Flies were treated with or without 200 μM RU486 for 14 days.
(B) Quantitation of WB depicted in (A) above. A separate experiment was run for each genotype. GFP expression was normalized to actin, for–RU and +RU samples, then expressed as a percentage of the average–RU value for each blot to enable comparison. 0N3R tau and C9orf72 (GR)100 significantly reduced GFP expression compared to -RU controls (* *p*<0.05 and ** *p*< 0.01 comparing +RU to–RU, student’s t-test). *p*>0.05 comparing +RU to–RU for the w1118 control line. Data are presented as means ± SEM and were analysed by student’s t-test for each genotype. N = 4 biological repeats of 10 fly heads per sample.
(C) ArcAβ42 did not alter mRNA expression of the cncC transcription factor, its binding partner MafS or Keap1. Data are presented as means ± SEM. *P*>0.05 comparing–RU to +RU for each gene (N = 4–5 repeats of 20 fly heads per sample; student’s t-test).
(TIF)

S4 Fig Keap1 loss of function effects on WT Aβ42-induced negative geotaxis. Heterozygous loss of Keap1 ameliorated climbing deficiency in flies expressing high levels of WT Aβ42 [37]. *P*<0.05 comparing +RU, Keap1 del or +RU Keap1 EY5 flies to +RU alone (two-way ANOVA and Tukey’s post-hoc comparison). N = 45–60 flies per condition analysed as 3–4 biological repeats of 15 flies.
(TIF)

S5 Fig Lithium dose-dependently activates cncC target genes, but its protection against Aβ42 toxicity is not cncC-dependent. (A) Venn diagrams showing overlap of GO categories differentially altered by lithium treatment of Aβ42 flies (+RU, +Li vs +RU) compared to cncC activation [38], most of which were up- or down-regulated in both conditions. Significant overlap was observed between categories up-regulated in lithium-treated Aβ42 flies and down-regulated by cncC activation. No significant overlap was observed between GO categories up-regulated by lithium treatment of Aβ42 flies and down-regulated by cncC.
(B) Lithium treatment increased mRNA levels of gstD2, a cncC target gene, as measured by quantitative PCR (qPCR). Data are presented as means ± SEM. *P<0.05 comparing +RU, Li 50 mM to -RU and +RU-treated elavGS>UAS-Arc Aβ42 flies (one-way ANOVA and Tukey’s HSD). N = 4 replicates of 20 fly heads per condition.
(C) Maximal lifespan extension of Aβ42-expressing flies by lithium (*p*<0.001 comparing +RU to +RU, +LiCl 50 mM) was unaltered by reducing cncC activity. *P* = 0.306 comparing +RU + LiCl 50mM to +RU + LiCl 50 mM, cncCK6 (log-rank test). N = 90–100 flies per condition.
(D) Comparison of effects of chronic or late lithium administration on Aβ42 toxicity. Aβ42 was induced by treatment with RU486 for 10 days, before switching to–RU medium, and lithium administered either from the point of induction (+RU [0-10d], +LiCl 25 mM [0-49d]) or following the induction period (+RU [0-10d], +LiCl 25 mM [10-49d]). Chronic treatment was required to exert protection against Aβ42-mediated climbing dysfunction. *P*<0.05 comparing +RU [0-10d] to +RU [0-10d], +LiCl 25 mM [0-49d]. *P*>0.05 comparing +RU [0-10d] to +RU [0-10d], +LiCl 25 mM [10-49d]. Data are presented as mean PI ± SEM and were analysed by two-way ANOVA and Tukey’s HSD post-hoc analyses. N = 4 biological repeats of 15 flies per condition.
(E) Consistent with reported effects on translation [47], lithium reduced Aβ42 peptide levels at early time-points following RU486 induction. Data are presented as means ± SEM. **p<0.01 and ***p<0.001 comparing +RU, +LiCl 25 mM and +RU, +LiCl 50 mM to +RU at both 2 and 7 days of age (two-way ANOVA and Tukey’s HSD). N = 5 biological repeats of 5 flies per condition.
(TIF)

S6 Fig Proteasome inhibitor effects on loss of Keap1-induced Aβ42 degradation. (A) Proteasome activity was measured using the fluorogenic peptide substrate LLVY-AMC (see methods). Enhanced activity in response to heterozygous Keap1^del^ mutation (* *p*<0.05 comparing +RU to +RU, Keap1^del^) was reduced to basal levels in ArcAβ42-expressing flies following treatment with 5–10 μM of the proteasome inhibitor Bortezomib for 14 days. A non-significant trend to inhibition was observed comparing +RU, +5 μM Keap1 del to +RU, Keap1 del flies. * *p*<0.05 comparing +RU, +10 μM Keap1 del to +RU, Keap1 del flies. Bortezomib had no effect on basal proteasome activity in control ArcAβ42-expressing flies in the absence of Keap^del^ (*P*>0.05). Data are presented as mean activities (pmoles/min/mg protein) ± SEM and were analysed by two-way ANOVA and Tukey’s HSD. N = 6 repeats of 10 fly heads per condition from two independent experiments.
(B) Total Aβ42 peptide levels following Bortezomib treatment of ArcAβ42 flies with or without heterozygous loss of Keap1 (Keap1^del^ and Keap1^EY5^). High concentrations of Bortezomib (>8 μM) reduced Aβ42 levels compared with untreated controls (* *p*<0.05 comparing +RU to +RU, + 8 μM Bortezomib for all genotypes). At doses of Bortezomib minimally required to suppress loss of Keap1-dependent proteasome activation without independently reducing Aβ42 levels (4–6 μM), degradation of Aβ42 in heterozygous Keap1^del^ and ^EY5^ flies was not prevented. *** *p*<0.001 comparing Keap1^del^ and Keap1^EY5^ to controls on all Bortezomib treatment conditions. Data are presented as means ± SEM and were analysed by two-way ANOVA and Tukey’s HSD. N = 4 repeats of 5 fly heads per condition.
(TIF)

S7 Fig Compound dose-response curves for Nrf2 activation. Nrf2 activity, as measured by induction of the target gene NQO1 (see methods), in response to compound treatment in WT SH-SY5Y cells.
Full dose-response curves are depicted for (A) the Nrf2 activator CDDO-Me, (B) the Keap1-Nrf2 disruptor 22h and (C) the GSK-3 inhibitor TDZD-8. TDZD-8 was a poor activator of Nrf2, inducing NQO1 only at 1 μM, and exerted toxic effects above this concentration (see Fig 6B).
(D) Log dose-response curves for Nrf2 activation using CDDO-Me and 22h. EC_50_ for NQO1 induction was 12.9 nM for CDDO-Me and 11.46 μM for 22h. Drug doses within range of these concentrations were used for comparative analysis of their effects in protecting against Aβ42 oligomer toxicity by activating Nrf2 (see Fig 6B and methods).
(TIF)

S8 Fig Aβ oligomer effects on Nrf2 and Keap1 protein levels. (A) Representative confocal images of Nrf2 (red), Keap1 (green) and DAPI (blue) immunostaining in Aβ oligomer (7PA2CM) vs wtCM-treated SH-SY5Y cells (see methods). Scale bar = 50 μM.
(B) Fluorescence intensity measurements of DAPI, Nrf2 and Keap1 staining across profile lines, indicated by arrows in (A), using Zen imaging software (Zeiss). Imaging indicates increased levels of Nrf2 and Keap1 proteins following Aβ oligomer treatment.
(C) Whole-cell immunostaining intensities were quantified in response to Aβ oligomers using IN Cell image analysis software (see methods). Error bars represent the standard error of the mean of five wells each containing 250 cells. * *P* = 0.011 comparing 7PA2CM to wtCM for Nrf2 and *p* = 0.014 comparing 7PA2CM to wtCM for Keap1 (student’s t-test).
(TIF)
