# Supplementary figures and images for: Direct Keap1-Nrf2 disruption as a potential therapeutic target for Alzheimer’s disease
Source: PLoS Genet. 2017 Mar 2;13(3):e1006593. doi: 10.1371/journal.pgen.1006593 (PMC5333801; doi:10.1371/journal.pgen.1006593)

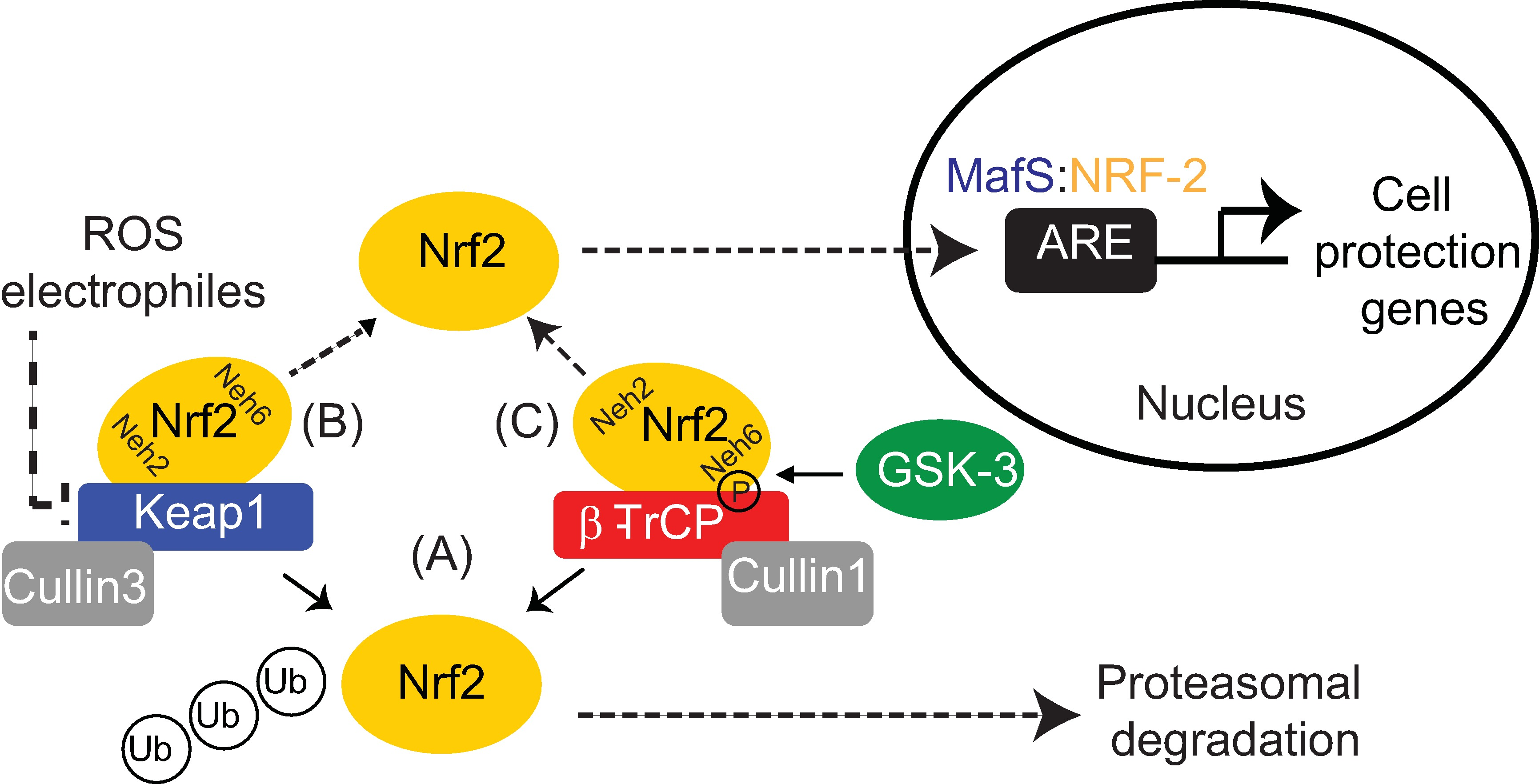

Supplement: S1 Fig — Under basal conditions (A) Keap1 binds to, and sequesters, Nrf-2 in the cytoplasm and actively targets it for ubiquitination by a Cullin3-based E3 ligase complex, thus enabling its degradation by the proteasome[1]. During conditions of oxidative or xenobiotic damage (B), Keap1 is inhibited by reactive oxidant species (ROS) and electrophiles, thus facilitating the stabilisation of Nrf-2 and enabling its translocation to the nucleus. Nrf-2 then forms dimers with small Maf (musculo-aponeurotic fibrosarcoma oncogene) proteins, which subsequently bind to and activate transcription of antioxidant response element (ARE)–containing cell protective genes. Alternatively, GSK-3 can inhibit Nrf-2 (C) by targeting it for ubiquitination by a β-TrcP-Cullin1 complex[43], through mechanisms that are independent of Keap1. A dual-degradation model for regulation of Nrf-2 under different pathophysiological conditions has been hypothesized[25]. (TIF) [file pgen.1006593.s001.tif]

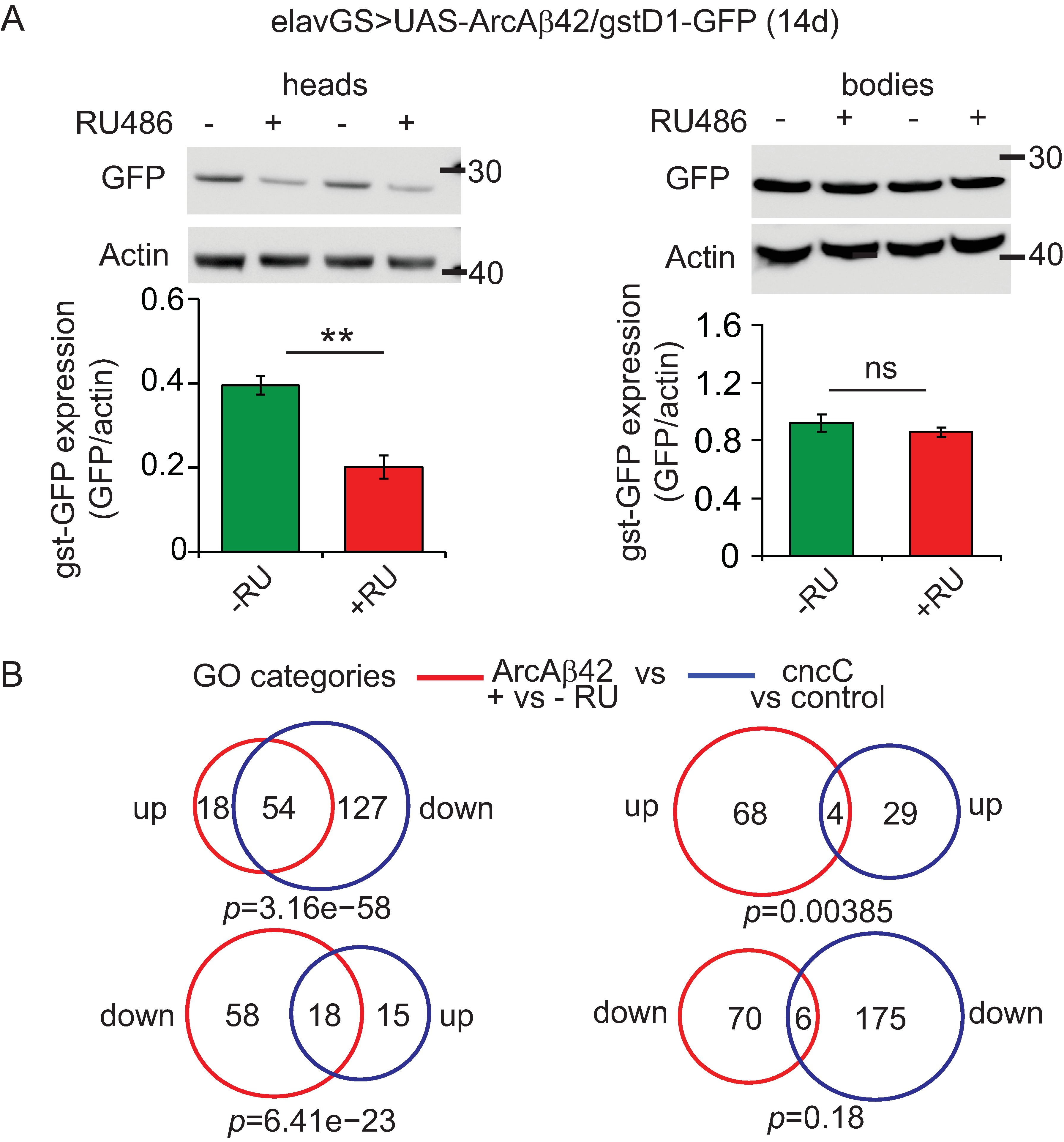

Supplement: S2 Fig — (A) cncC activity was measured in 14-day-old Arc Aβ42-expressing fly heads and bodies. ** p<0.01 comparing +RU to–RU in heads. For bodies only, no significant effect of ArcAβ42 on gstD1(ARE)–GFP reporter expression was observed compared to controls (p>0.05 comparing +RU to–RU controls, student’s t-test). Data represent means ± SEM. N = 4 replicates of 10 flies per condition. (B) Venn diagrams showing the overlap of GO categories differentially altered by Aβ42 and cncC[38], most of which were reciprocally regulated. Few GO terms were significantly up-regulated by both Aβ42 and cncC activation, and no significant overlap was observed between genes down-regulated in both conditions. (TIF) [file pgen.1006593.s002.tif]

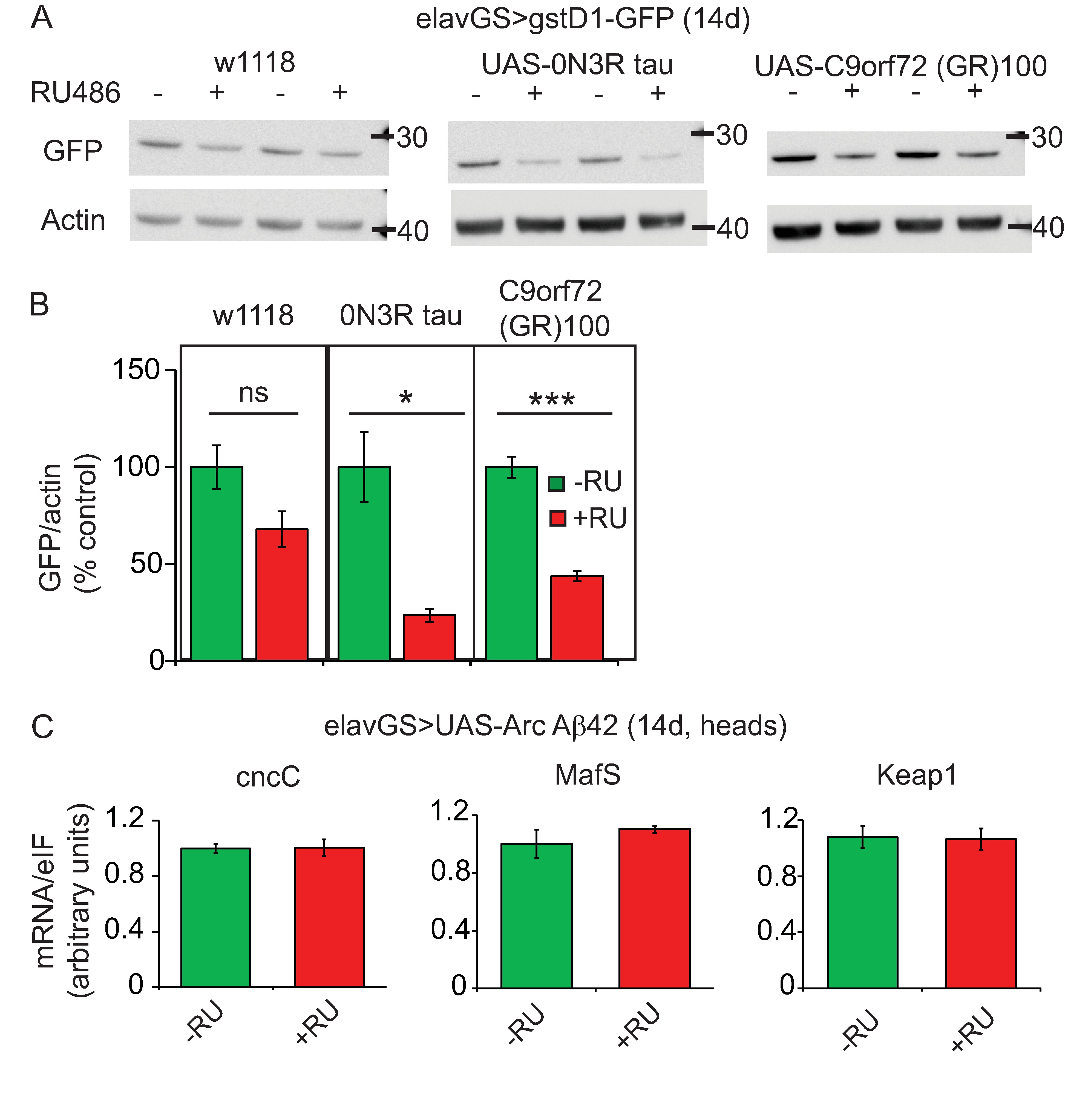

Supplement: S3 Fig — (A) Western blot analysis of gstD1(ARE)–GFP reporter expression in w1118 controls and flies over-expressing human 0N3R tau or C9orf72 (GR)100 DPRs in adult neurons. Flies were treated with or without 200 μM RU486 for 14 days. (B) Quantitation of WB depicted in (A) above. A separate experiment was run for each genotype. GFP expression was normalized to actin, for–RU and +RU samples, then expressed as a percentage of the average–RU value for each blot to enable comparison. 0N3R tau and C9orf72 (GR)100 significantly reduced GFP expression compared to -RU controls (* p<0.05 and ** p< 0.01 comparing +RU to–RU, student’s t-test). p>0.05 comparing +RU to–RU for the w1118 control line. Data are presented as means ± SEM and were analysed by student’s t-test for each genotype. N = 4 biological repeats of 10 fly heads per sample. (C) ArcAβ42 did not alter mRNA expression of the cncC transcription factor, its binding partner MafS or Keap1. Data are presented as means ± SEM. P>0.05 comparing–RU to +RU for each gene (N = 4–5 repeats of 20 fly heads per sample; student’s t-test). (TIF) [file pgen.1006593.s003.tif]

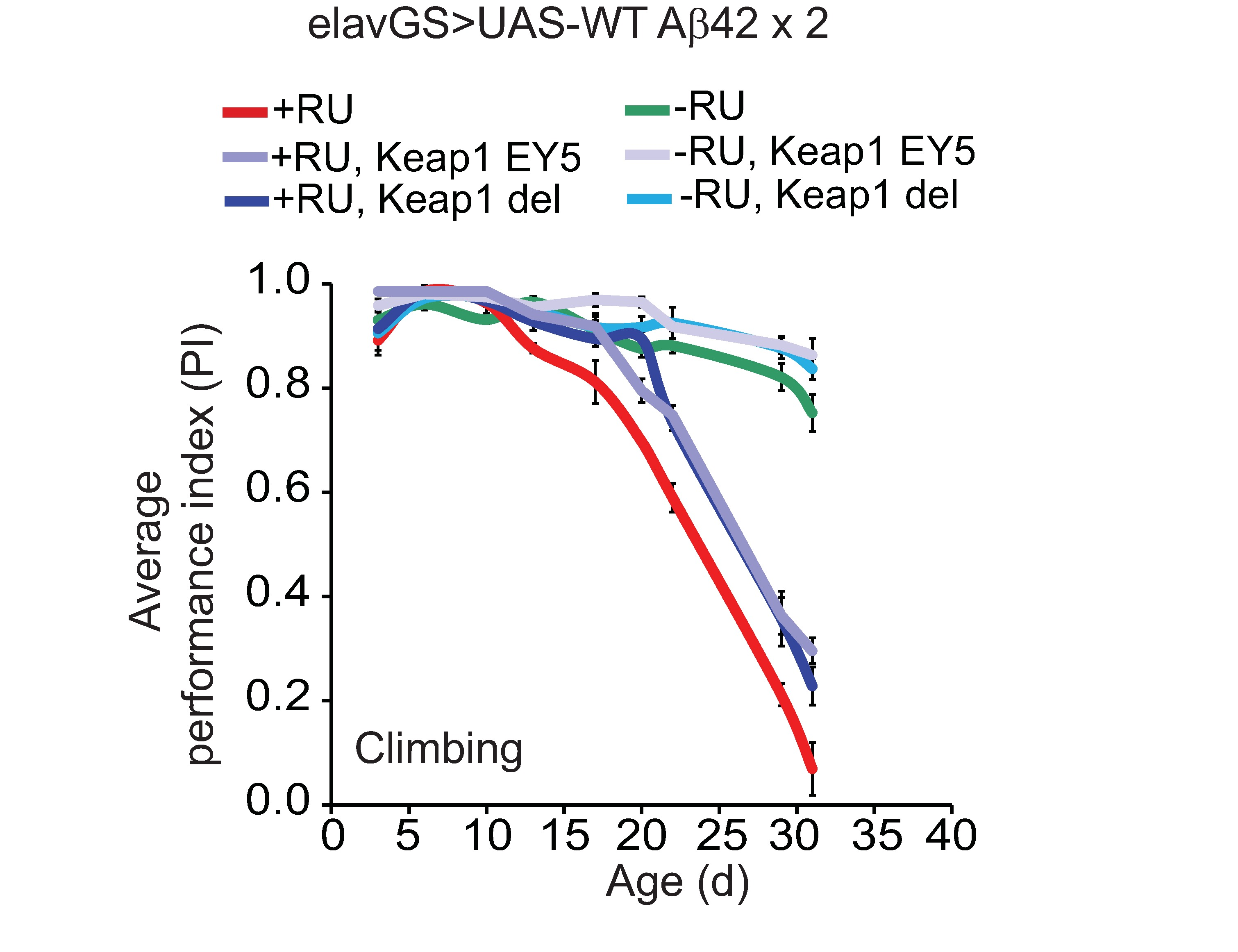

Supplement: S4 Fig — Heterozygous loss of Keap1 ameliorated climbing deficiency in flies expressing high levels of WT Aβ42 [37]. P<0.05 comparing +RU, Keap1 del or +RU Keap1 EY5 flies to +RU alone (two-way ANOVA and Tukey’s post-hoc comparison). N = 45–60 flies per condition analysed as 3–4 biological repeats of 15 flies. (TIF) [file pgen.1006593.s004.tif]

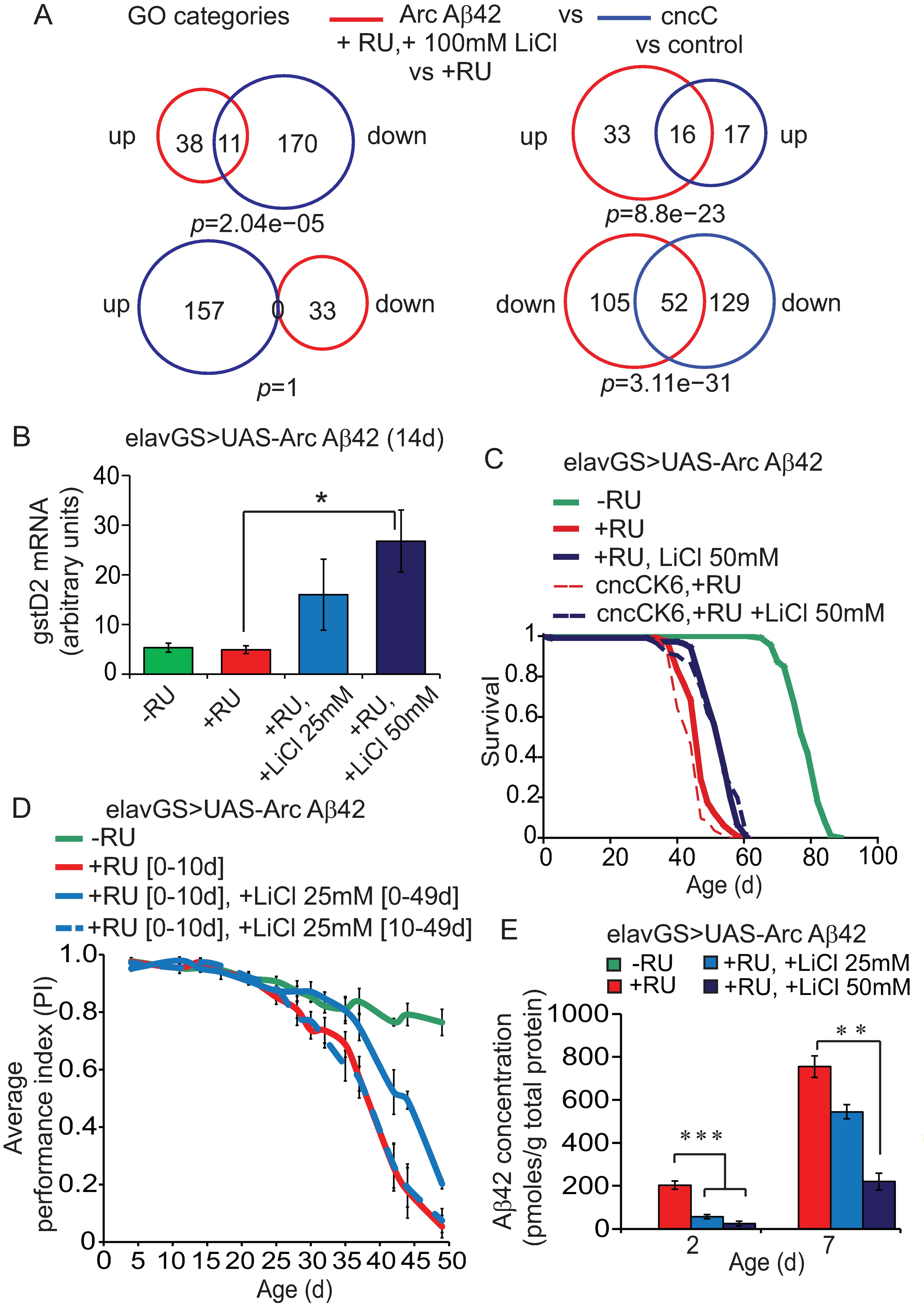

Supplement: S5 Fig — (A) Venn diagrams showing overlap of GO categories differentially altered by lithium treatment of Aβ42 flies (+RU, +Li vs +RU) compared to cncC activation [38], most of which were up- or down-regulated in both conditions. Significant overlap was observed between categories up-regulated in lithium-treated Aβ42 flies and down-regulated by cncC activation. No significant overlap was observed between GO categories up-regulated by lithium treatment of Aβ42 flies and down-regulated by cncC. (B) Lithium treatment increased mRNA levels of gstD2, a cncC target gene, as measured by quantitative PCR (qPCR). Data are presented as means ± SEM. *P<0.05 comparing +RU, Li 50 mM to -RU and +RU-treated elavGS>UAS-Arc Aβ42 flies (one-way ANOVA and Tukey’s HSD). N = 4 replicates of 20 fly heads per condition. (C) Maximal lifespan extension of Aβ42-expressing flies by lithium (p<0.001 comparing +RU to +RU, +LiCl 50 mM) was unaltered by reducing cncC activity. P = 0.306 comparing +RU + LiCl 50mM to +RU + LiCl 50 mM, cncCK6 (log-rank test). N = 90–100 flies per condition. (D) Comparison of effects of chronic or late lithium administration on Aβ42 toxicity. Aβ42 was induced by treatment with RU486 for 10 days, before switching to–RU medium, and lithium administered either from the point of induction (+RU [0-10d], +LiCl 25 mM [0-49d]) or following the induction period (+RU [0-10d], +LiCl 25 mM [10-49d]). Chronic treatment was required to exert protection against Aβ42-mediated climbing dysfunction. P<0.05 comparing +RU [0-10d] to +RU [0-10d], +LiCl 25 mM [0-49d]. P>0.05 comparing +RU [0-10d] to +RU [0-10d], +LiCl 25 mM [10-49d]. Data are presented as mean PI ± SEM and were analysed by two-way ANOVA and Tukey’s HSD post-hoc analyses. N = 4 biological repeats of 15 flies per condition. (E) Consistent with reported effects on translation [47], lithium reduced Aβ42 peptide levels at early time-points following RU486 induction. Data are presented as means ± SEM. **p<0.01 and ***p<0.001 comparing [file pgen.1006593.s005.tif]

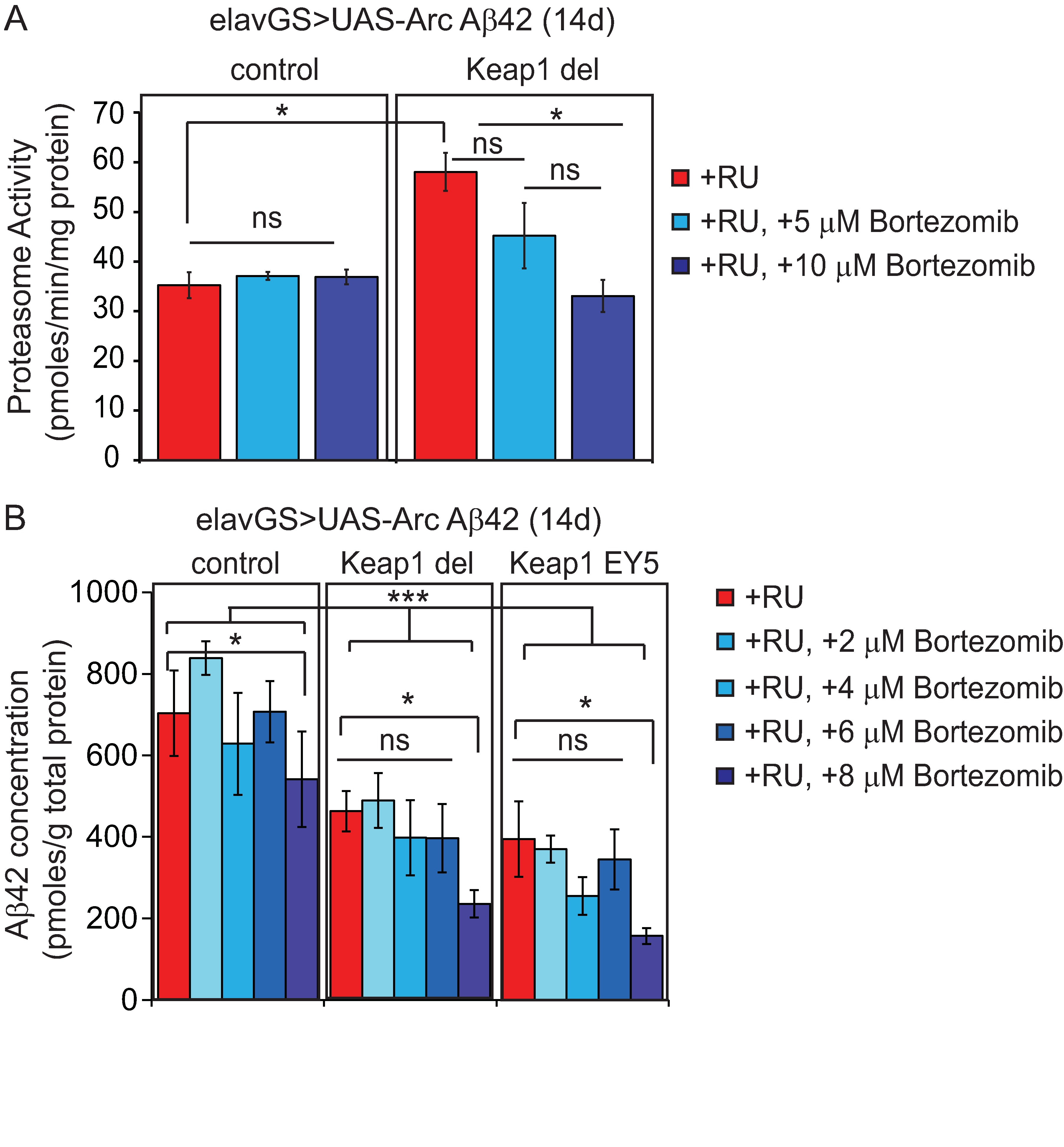

Supplement: S6 Fig — (A) Proteasome activity was measured using the fluorogenic peptide substrate LLVY-AMC (see methods). Enhanced activity in response to heterozygous Keap1del mutation (* p<0.05 comparing +RU to +RU, Keap1del) was reduced to basal levels in ArcAβ42-expressing flies following treatment with 5–10 μM of the proteasome inhibitor Bortezomib for 14 days. A non-significant trend to inhibition was observed comparing +RU, +5 μM Keap1 del to +RU, Keap1 del flies. * p<0.05 comparing +RU, +10 μM Keap1 del to +RU, Keap1 del flies. Bortezomib had no effect on basal proteasome activity in control ArcAβ42-expressing flies in the absence of Keapdel (P>0.05). Data are presented as mean activities (pmoles/min/mg protein) ± SEM and were analysed by two-way ANOVA and Tukey’s HSD. N = 6 repeats of 10 fly heads per condition from two independent experiments. (B) Total Aβ42 peptide levels following Bortezomib treatment of ArcAβ42 flies with or without heterozygous loss of Keap1 (Keap1del and Keap1EY5). High concentrations of Bortezomib (>8 μM) reduced Aβ42 levels compared with untreated controls (* p<0.05 comparing +RU to +RU, + 8 μM Bortezomib for all genotypes). At doses of Bortezomib minimally required to suppress loss of Keap1-dependent proteasome activation without independently reducing Aβ42 levels (4–6 μM), degradation of Aβ42 in heterozygous Keap1del and EY5 flies was not prevented. *** p<0.001 comparing Keap1del and Keap1EY5 to controls on all Bortezomib treatment conditions. Data are presented as means ± SEM and were analysed by two-way ANOVA and Tukey’s HSD. N = 4 repeats of 5 fly heads per condition. (TIF) [file pgen.1006593.s006.tif]

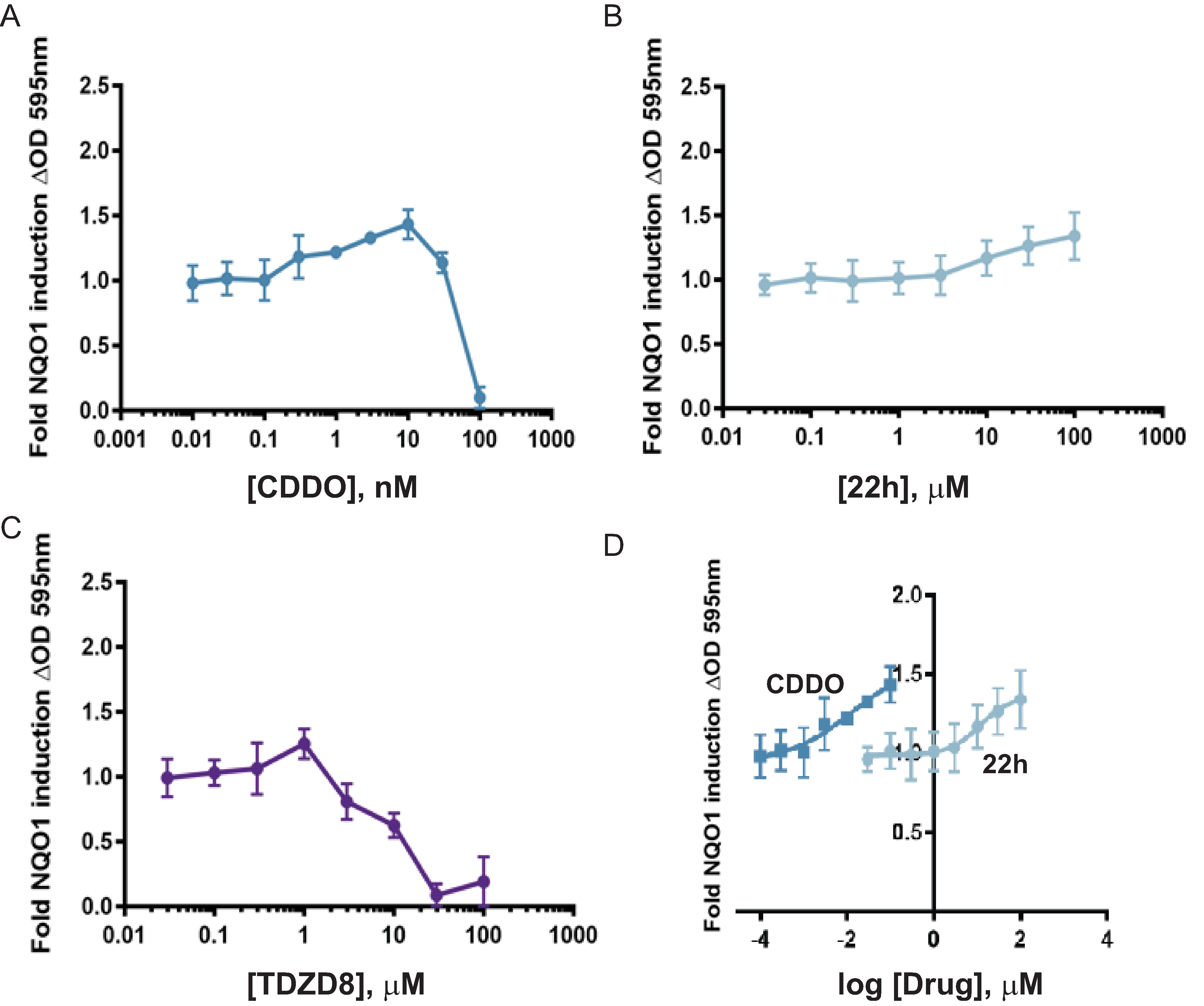

Supplement: S7 Fig — Nrf2 activity, as measured by induction of the target gene NQO1 (see methods), in response to compound treatment in WT SH-SY5Y cells. Full dose-response curves are depicted for (A) the Nrf2 activator CDDO-Me, (B) the Keap1-Nrf2 disruptor 22h and (C) the GSK-3 inhibitor TDZD-8. TDZD-8 was a poor activator of Nrf2, inducing NQO1 only at 1 μM, and exerted toxic effects above this concentration (see Fig 6B). (D) Log dose-response curves for Nrf2 activation using CDDO-Me and 22h. EC50 for NQO1 induction was 12.9 nM for CDDO-Me and 11.46 μM for 22h. Drug doses within range of these concentrations were used for comparative analysis of their effects in protecting against Aβ42 oligomer toxicity by activating Nrf2 (see Fig 6B and methods). (TIF) [file pgen.1006593.s007.tif]

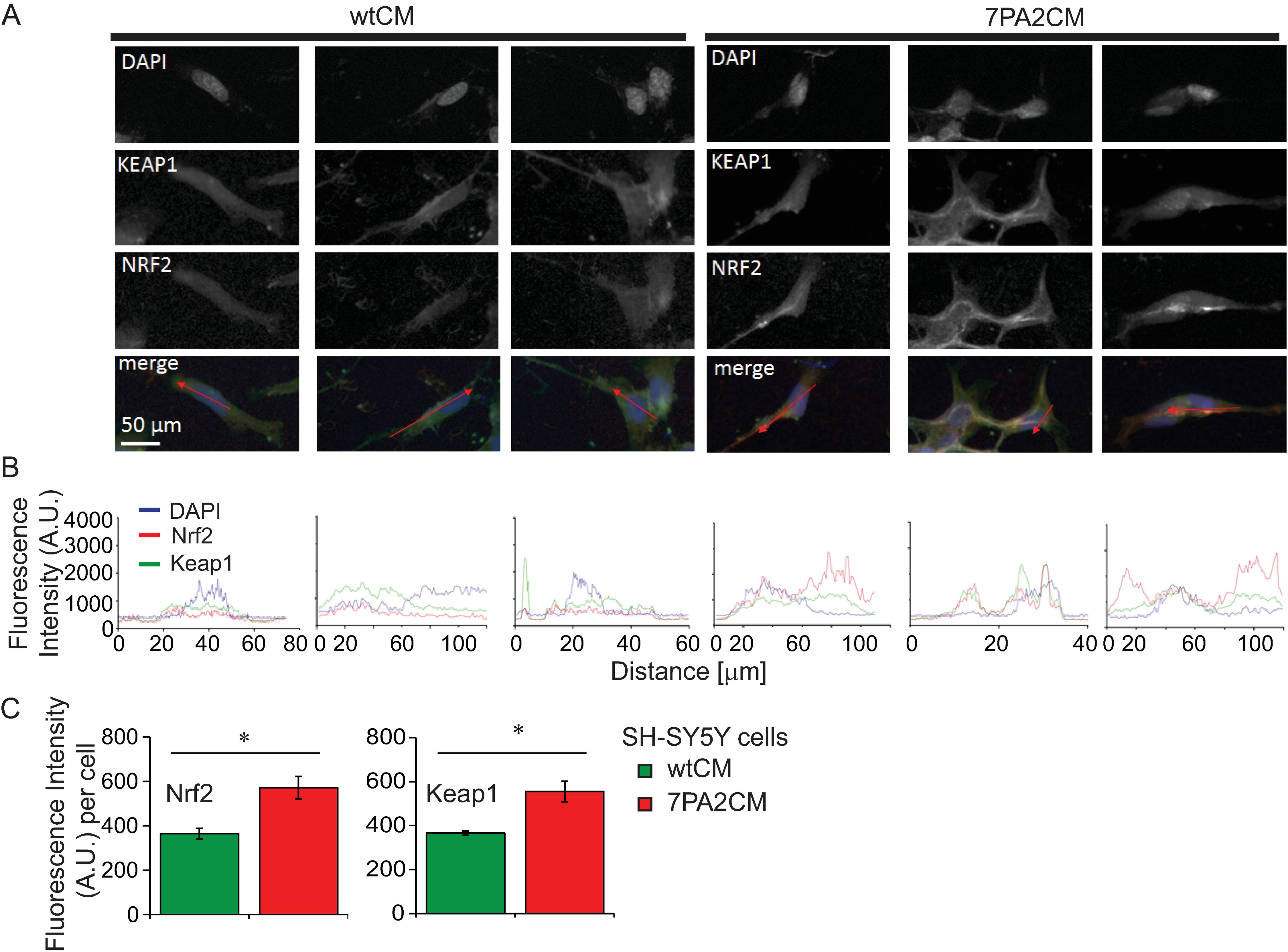

Supplement: S8 Fig — (A) Representative confocal images of Nrf2 (red), Keap1 (green) and DAPI (blue) immunostaining in Aβ oligomer (7PA2CM) vs wtCM-treated SH-SY5Y cells (see methods). Scale bar = 50 μM. (B) Fluorescence intensity measurements of DAPI, Nrf2 and Keap1 staining across profile lines, indicated by arrows in (A), using Zen imaging software (Zeiss). Imaging indicates increased levels of Nrf2 and Keap1 proteins following Aβ oligomer treatment. (C) Whole-cell immunostaining intensities were quantified in response to Aβ oligomers using IN Cell image analysis software (see methods). Error bars represent the standard error of the mean of five wells each containing 250 cells. * P = 0.011 comparing 7PA2CM to wtCM for Nrf2 and p = 0.014 comparing 7PA2CM to wtCM for Keap1 (student’s t-test). (TIF) [file pgen.1006593.s008.tif]
